# Targeted Repair of Vascular Injury by Adipose‐Derived Stem Cells Modified with P‐Selectin Binding Peptide

**DOI:** 10.1002/advs.201903516

**Published:** 2020-04-22

**Authors:** Hongyu Yan, Xingyan Mi, Adam C. Midgley, Xinchen Du, Ziqi Huang, Tingting Wei, Ruihua Liu, Tengzhi Ma, Dengke Zhi, Dashuai Zhu, Ting Wang, Guowei Feng, Ying Zhao, Weiye Zhang, Ju He, Meifeng Zhu, Deling Kong, Kai Wang

**Affiliations:** ^1^ Key Laboratory of Bioactive Materials Ministry of Education College of Life Sciences Nankai University Tianjin 300071 China; ^2^ School of Medicine Nankai University Tianjin 300071 China; ^3^ Urban Transport Emission Control Research Centre College of Environmental Science and Engineering Nankai University Tianjin 300071 China; ^4^ Department of Genitourinary Oncology Tianjin Medical University Cancer Institute and Hospital National Clinical Research Center for Cancer Key Laboratory of Cancer Prevention and Therapy Tianjin 300060 China; ^5^ Donation Services Tianjin First Central Hospital Tianjin 300192 China; ^6^ Department of Vascular Surgery Tianjin First Central Hospital Tianjin 300192 China

**Keywords:** adipose‐derived stem cells, living cell‐surface modification, PEG lipid, P‐selectin, vascular injury

## Abstract

Percutaneous coronary intervention for coronary artery disease treatment often results in pathological vascular injury, characterized by P‐selectin overexpression. Adipose‐derived stem cells (ADSCs) therapeutic efficacy remains elusive due to poor ADSCs targeting and retention in injured vessels. Here, conjugated P‐selectin binding peptide (PBP) to polyethylene glycol‐conjugated phospholipid derivative (DMPE‐PEG) linkers (DMPE‐PEG‐PBP; DPP) are used to facilitate the modification of PBP onto ADSCs cell surfaces via hydrophobic interactions between DMPE‐PEG and the phospholipid bilayer. DPP modification neither has influence on ADSCs proliferation nor apoptosis/paracrine factor gene expression. A total of 5 × 10^−6^ m DPP‐modified ADSCs (DPP‐ADSCs) strongly binds to P‐selectin‐displaying activated platelets and endothelial cells (ECs) in vitro and to wire‐injured rat femoral arteries when administered by intra‐arterial injection. Targeted binding of ADSCs shields injury sites from platelet and leukocyte adhesion, thereby decreasing inflammation at injury sites. Furthermore, targeted binding of ADSCs recovers injured ECs functionality and reduces platelet‐initiated vascular smooth muscle cells (VSMCs) chemotactic migration. Targeted binding of DPP‐human ADSCs to balloon‐injured human femoral arteries is also demonstrated in ex vivo experiments. Overall, DPP‐ADSCs promote vascular repair, inhibit neointimal hyperplasia, increase endothelium functionality, and maintain normal VSMCs alignment, supporting preclinical noninvasive utilization of DPP‐ADSCs for vascular injury.

## Graphical abstract

The cell modification approach, utilizing hydrophobic interaction between amphiphilic molecules (polyethylene glycol‐conjugated phospholipid‐derivative, DMPE‐PEG) and adipose‐derived stem cells (ADSCs) membrane, is presented. The approach is facile, time‐efficient, and noncytotoxic. Systemically administered DMPE‐PEG‐P‐selectin binding peptide modified ADSCs (DPP‐ADSCs) exhibit significantly increased targeting ability to vascular injuries. This study demonstrates advantageous mechanisms of repair, providing new preclinical avenues for cardiovascular disease treatment.

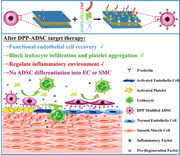

## 1 Introduction

Cardiovascular diseases, including coronary artery occlusions, are the leading cause of mortality worldwide.^[^ ^1^ ^]^ The predominant surgical intervention for occluded coronary and peripheral arteries is percutaneous coronary intervention (PCI), which comprises balloon angioplasty and endoluminal stenting techniques.^[^ ^2^ ^]^ These surgical methods frequently result in incidental vascular wall injuries, such as endothelium denudation and barotrauma. Endothelium denudation exposes the thrombogenic subendothelial matrix, which adheres to and activates circulating platelets; whereas barotrauma results in endothelial cell (EC) activation.^[^ ^3^ ^]^ Activated platelets and ECs induce the binding of leukocytes and platelets, which increases the risk of developing pathological vascular changes such as thrombus formation, inflammation, restenosis and reemergence of arterial occlusion.^[^ ^4^ ^]^

The selectin family (L‐selectin, E‐selectin, and P‐selectin) is all involved in regulating pathological vascular changes but having different expression at the vascular injury sites. L‐selectin is exclusively expressed on leukocyte surfaces. Activation of ECs results in de novo synthesis of E‐selectin, reaching peak cell‐surface expression by 4 h.^[^ ^5^ ^]^ P‐selectin is stored within platelet *α*‐granules and ECs Weibel–Palade bodies. Within minutes of activation, P‐selectin translocates to the external plasma membrane in a protein‐synthesis‐independent manner.^[^ ^6^ ^]^ In contrast to the delayed E‐selectin cell‐surface presentation, the rapid and prominent expression of P‐selectin on both activated ECs and platelets^[^ ^7^ ^]^ makes it an appealing candidate for targeting injured arteries.

Rapid induction of P‐selectin cell‐surface expression in activated platelet and ECs drives ancillary mechanisms of inter‐platelet and platelet–ECs interactions, promoting thrombus formation.^[^ ^8^ ^]^ P‐selectin also promotes recruitment of circulating leukocytes, subsequently leading to elevated release of proinflammatory cytokines, such as interleukin (IL)‐1 and IL‐6.^[^ ^9^ ^]^ This, in conjunction with other factors released from activated platelets and ECs, such as platelet‐derived growth factor and monocyte chemotactic protein‐1, induces migration and proliferation of vascular smooth muscle cells (VSMCs) and promotes arterial restenosis and occlusion.^[^ ^10^ ^]^ Thus, masking injured vessel P‐selectin overexpression may inhibit pathological vascular changes.

Adipose‐derived stem cells (ADSCs) are a popularized cell source for use in tissue engineering and regenerative medicine due to their abundance, ease of isolation, self‐renewal properties, and ethical nature.^[^ ^11^ ^]^ Following administration to injured sites, ADSCs are thought to enhance tissue repair and regeneration through two main processes: i) ADSCs secretion of bioactive factors, which affect the surrounding microenvironment to promote angiogenesis, regulate inflammation, and enhance tissue repair;^[^ ^12^ ^]^ ii) ADSCs differentiation into various cell phenotypes (including ECs, myocytes, neurocytes, etc.), which may result in integration into host tissues.^[^ ^13^ ^]^

A major challenge facing stem cell therapeutics is the efficient delivery of cells to tissues of interest.^[^ ^14^ ^]^ Local injection into tissues or infusion into proximal blood vessels represents two potential delivery routes. For vascular injuries, local injection is hazardous as arterial walls are thin and pulsatile. Intra‐arterial infusion is minimally invasive, enables repetitive dosing, and circumvents problems associated with secondary vascular injury and calcification. Many reports have shown that cell‐surface modification with bioactive molecules with high affinity to pathological markers can improve the efficiency of intravascular‐delivered cells to target tissues of interest and to benefit tissue repair. A previous study demonstrated that systemically injected ADSCs could recruit to injured vessels.^[^ ^15^ ^]^ However, ADSCs lack surface ligands with high affinity to P‐selectin.^[^ ^16^ ^]^ Thus, modifiying bioactive molecular with binding specificity for P‐selectin on the ADSCs surface may enhance their targeting to vascular injury sites.

Recently, methods have been developed to facilitate cell‐membrane display of exogenously added bioactive molecules that enhance cell targeting to activated ECs and their molecular markers. These modifications include the conversion of mesenchymal stem cells (MSCs) CD44 receptors into E‐selectin ligands through *α*‐1,3‐fucosyltransferase catalysis;^[^ ^17^ ^]^ palmitated protein‐G enabled cell‐surface coating with intercellular cell adhesion molecule‐1 (ICAM‐1) antibody;^[^ ^18^ ^]^ and avidin‐biotin covalent coupling of SLeX (P‐selectin glycoprotein ligand‐1 active site from leukocytes) onto MSCs membranes.^[^ ^19^ ^]^ Despite the demonstrated success of these approaches, simplistic and rapidly reproducible methods that avoid the disadvantages of gene modification,^[^ ^20^ ^]^ restrictive enzyme catalysis sites,^[^ ^21^ ^]^ the use of potentially immunogenic modifications (streptavidin),^[^ ^22^ ^]^ and the cell toxicity induced by covalent coupling^[^ ^23^ ^]^ would be favorable for clinical application.

Polyethylene glycol‐conjugated phospholipid (lipid‐PEG) linkers are useful tools to anchor bioactive molecules to cell membranes, via hydrophobic interactions between lipid‐PEG and the phospholipid bilayer. Lipid‐PEG provides a biocompatible and attractive solution for modifying cells to display PEG‐conjugatable receptor‐specific antibodies, single‐stranded DNA, and peptides on their surfaces.^[^ ^24^ ^]^ For example, Iwata's group immobilized human soluble complement receptor 1 and heparin onto islet cells using maleimide‐PEG‐phospholipids, which provided anti‐complement and anticoagulation activities.^[^ ^25^ ^]^ This simplistic and mild method did not affect islet cell function. Modified islet cells prevented early islet graft loss, resulting in the reversal of hyperglycemia. Won et al., successfully anchored CXC chemokine receptor 4 (CXCR4) onto MSCs using CXCR4‐PEG‐lipid, which enhanced chemotaxis towards serum‐derived factor 1 gradients.^[^ ^26^ ^]^

Targeting of P‐selectin has been achieved through the use of antibodies^[^ ^27^ ^]^ and the peptide sequence, DAEWVDVS, which has a remarkably high affinity and binding specificity for P‐selectin.^[^ ^28^ ^]^ Polypeptide production is more economical than antibody production, and offers the advantage of high‐throughput yields obtained by a solid‐phase peptide synthesis techniques.^[^ ^29^ ^]^ The relatively simplistic and well‐defined chemical structure of polypeptides allows for ease of conjugation or modification according to given circumstances, while antibodies usually cannot be arbitrarily modified without impairing affinity.^[^ ^30^ ^]^ Therefore, we combined P‐selectin binding peptide (PBP) with polyethylene glycol‐conjugated phospholipid derivative (DMPE‐PEG), a commonly used lipid‐PEG, to facilitate the modification of PBP onto the surface of ADSCs. Bioavailability and biosafety of DMPE‐PEG‐PBP (DPP) modifications were evaluated in vitro and DPP‐modified ADSCs (DPP‐ADSCs) were optimized for in vivo testing and targeting to injured blood vessels. Further efforts were then made to reveal the underlying mechanisms of DPP‐ADSCs therapeutic effect on vascular injury repair.

## 2 Results

### 2.1 DPP Characterization

DPP was synthesized by the Michael addition reaction between the carbon–carbon double bonds of DMPE‐PEG‐maleimide (DMPE‐PEG‐MAL) and the thiol group of PBP (Figure S1a, Supporting Information). Analysis by ^1^H NMR (Figure S1b, Supporting Information) showed the absence of MAL (6.9 ppm) and the presence of the PBP indole ring (7.0–7.6 ppm region) in the spectra of DPP, indicating the successful synthesis of DPP.

### 2.2 Parameters of DPP Cell‐Surface Modification

**Figure 1:**
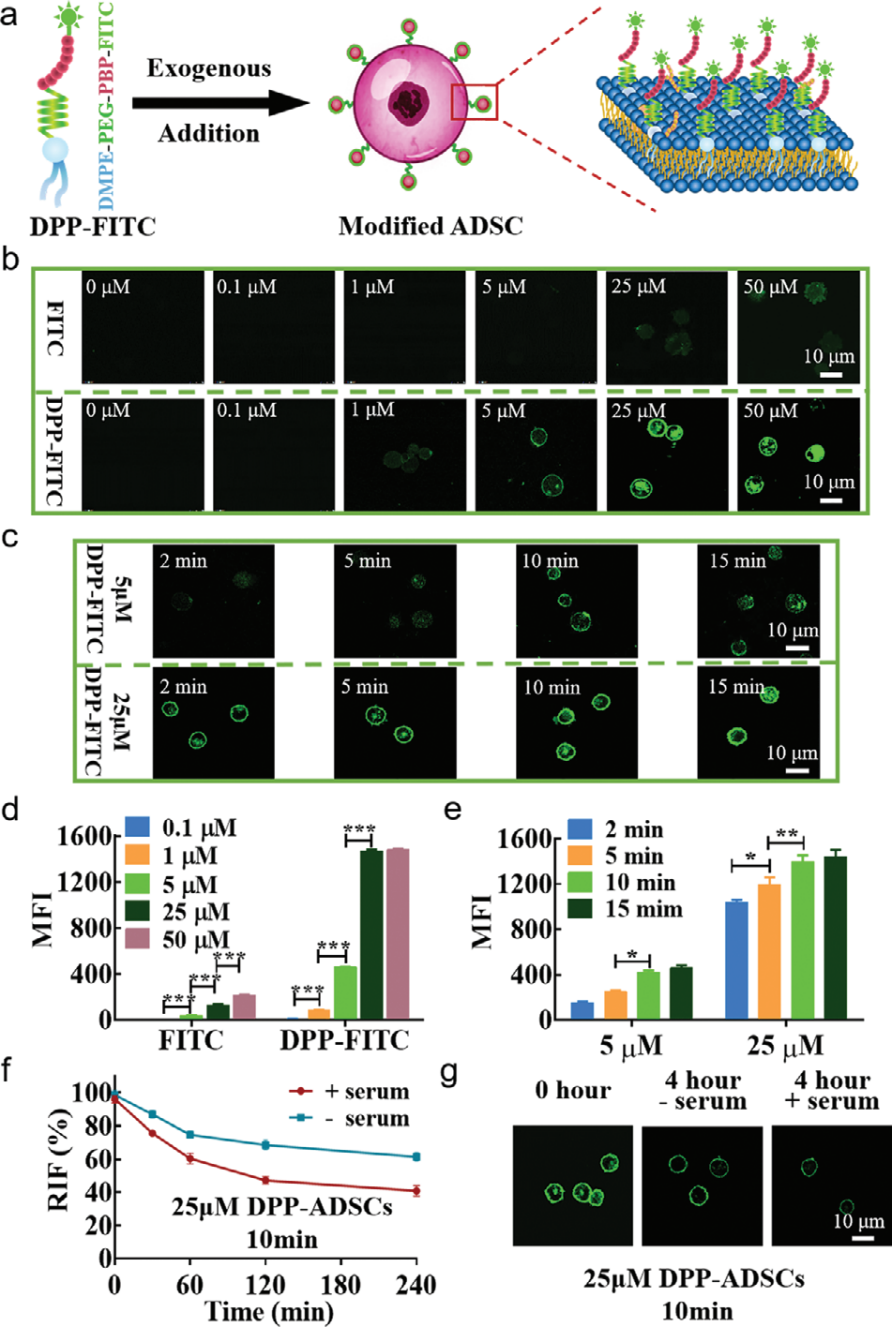
Optimization of incubation conditions on modification yield. a) Schematic diagram of ADSC modification by exogenous addition of DPP‐FITC, which results in ADSC cell surface display of DPP‐FITC. b) Representative confocal micrographs of ADSCs modified with different DPP‐FITC and FITC concentrations after 10 min incubation time (*n* = 3). c) Representative confocal micrographs of 5 × 10^−6^ and 25 × 10^−6^ m DPP‐FITC‐modified ADSCs at different incubation times (*n* = 3). d) MFI of ADSCs modified with different DPP‐FITC and FITC concentrations (*n* = 3). e) MFI of 5 × 10^−6^ and 25 × 10^−6^ m DPP‐FITC‐modified ADSCs at different incubation times (*n* = 3). ADSCs modified with FITC only were used as control groups. f) The relative fluorescence intensity (RFI) showed the kinetics of the DMPE‐PEG on the surface of ADSCs in the presence or the absence of serum. The MFI of as‐modified ADSCs was defined as 100% (*n* = 3). g) Confocal micrographs showed that DPP‐FITC could still be detected on the surface of ADSCs after incubation at 37 °C for 4 h, regardless of the presence of serum (*n* = 3).

As shown in the schematic (**Figure** **1**a), DPP‐fluorescein isothiocyanate isomer (FITC) was synthesized to visualize the cell‐surface modification. ADSCs assessed by flow cytometry (FCM) indicated that the majority were positive for mesenchymal markers: CD29 (99.6%), CD44 (99.4%), CD105 (99.7%), and CD90 (99.7%); and all were negative for CD31, CD34, and CD45 (Figure S2, Supporting Information). After incubation with different concentrations of FITC and DPP‐FITC for 10 min, ADSCs were assessed by confocal microscopy (Figure 1b) and FCM (Figure 1d). Confocal images showed that 1 × 10^−6^ m DPP could sufficiently cover the surfaces of 5 × 10^5^ ADSCs, whereas FITC alone required at least 25 × 10^−6^ m. The mean fluorescence intensity (MFI) of DPP‐FITC‐labeled ADSCs, as assessed by FCM, showed positive correlation with increasing DPP‐FITC concentration. MFI plateaued at 25 × 10^−6^ m DPP‐FITC and did not increase further when utilizing 50 × 10^−6^ m DPP‐FITC. These results indicated that cell surfaces were saturated with 25 × 10^−6^ m DPP. Subsequently, the fluorescence signal of ADSCs incubated with 5 × 10^−6^ and 25 × 10^−6^ m DPP‐FITC was assessed by confocal microscopy and FCM at the predetermined time points. The fluorescence of ADSCs modified with 5 × 10^−6^ m DPP‐FITC was lower than 25 × 10^−6^ m DPP‐FITC, at the same incubation time; and after 5 min of incubation time, ADSCs modified with 5 × 10^−6^ m DPP‐FITC were detectable by confocal microscopy (Figure 1c). FCM analysis showed that the MFI of ADSCs modified with 5 × 10^−6^ or 25 × 10^−6^ m DPP‐FITC at the 15 min time‐point was comparable with that at the 10 min time‐point (Figure 1e), which indicated that the modification reached saturation by 10 min. To investigate cell‐surface kinetics of DPP modification, ADSCs modified with 25 × 10^−6^ m DPP‐FITC for 10 min were incubated in 37 °C culture medium in the presence or absence of serum for up to 4 h. FCM showed that regardless of serum presence, the relative fluorescent intensity (RFI) of DPP‐FITC decreased gradually over time (Figure 1f). After 4 h, the RFI in the presence or the absence of serum was 40.91% ± 3.28% and 61.50% ± 2.68% compared to initial RFI, respectively; yet FITC signal remained detectable using confocal microscopy at consistent laser settings (Figure 1g). These data suggested that DPP remained on the surface of ADSCs at least for 4 h, regardless of serum presence.

### 2.3 DPP Modification Effect on ADSCs Behavior

**Figure 2:**
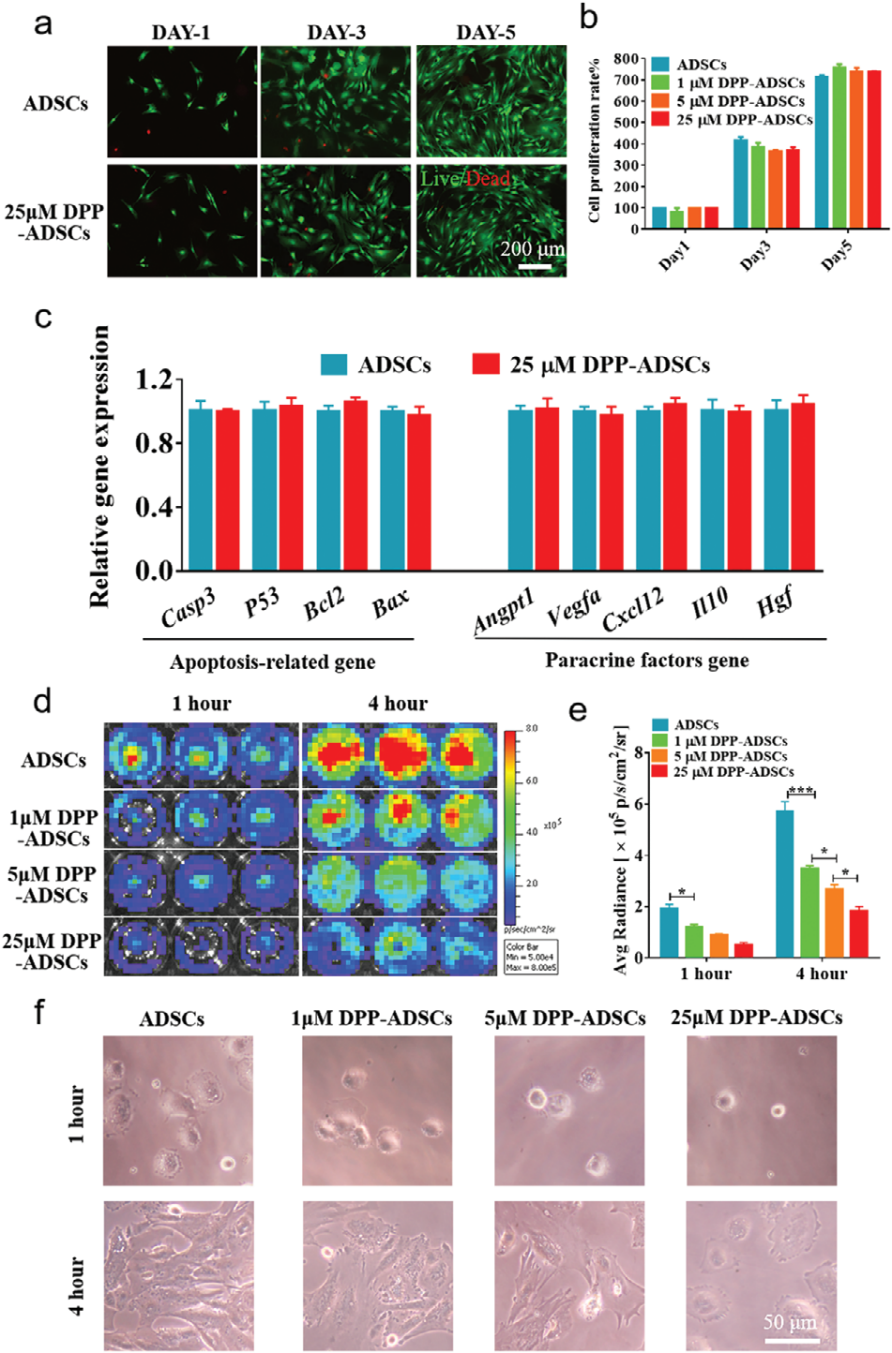
Effect of DPP modification on proliferation, gene expression, and adhesion of ADSCs. a) Representative Live/Dead fluorescence staining images of ADSCs and 25 × 10^−6^ m DPP‐ADSCs (*n* = 5). b) The proliferation of unmodified and DPP‐ADSCs was tested by CCK‐8 assays (*n* = 5). c) The relative expression of apoptosis‐related genes and paracrine factor genes, in ADSCs with (cyan bars) or without (red bars) 25 × 10^−6^ m DPP modification (*n* = 3). d) The adhesion of unmodified and DPP‐Luc‐ADSCs on TCPS was observed by BLI after incubation for 1 and 4 h (*n* = 4). e) The quantitative analysis of unmodified and DPP‐modified Luc‐ADSCs adhesion, based on the Luc fluorescence intensities. f) The phase‐contrast microscopy showed that there was no difference in cell morphology of unmodified and DPP‐modified ADSCs adhered on TCPS after 1 and 4 h incubation (*n* = 4).

ADSCs modified with 1 × 10^−6^, 5 × 10^−6^, and 25 × 10^−6^ m DPP for 10 min and unmodified ADSCs were cultured for 5 days. Live/Dead staining showed that ADSCs modified with all DPP concentrations maintained a steady growth state, exhibiting almost no cell death (**Figure** **2**a). CCK‐8 assays showed that modified ADSCs proliferation was comparable to unmodified ADSCs (Figure 2b). As shown in Figure 2c, the expression of apoptosis‐related genes (*Casp3*, *P53*, *Bcl2*, and *Bax*) and paracrine cytokines genes (*Angpt1*, *Vegfa*, *Cxcl12*, *Il10*, and *Hgf*) shows no discernable differences between unmodified and 25 × 10^−6^ m DPP‐ADSCs after culture for 24 h, which indicated that DPP modification did not affect survival or secretory behaviors of ADSCs. DPP modification also had no influence on ADSCs migration (Figure S3, Supporting Information). Inhibition of adhesion to tissue culture plastic substrate (TCPS) (Figure 2d,e) and collagen IV‐coated TCPS (collagen IV is a core component of the subendothelial matrix)^[^ ^31^ ^]^ (Figure S4a,b, Supporting Information) was observed, in a concentration‐dependent manner. Adhesiveness was decreased by 1 × 10^−6^, 5 × 10^−6^, and 25 × 10^−6^ mw DPP at 4 h to ≈61.14% ± 3.20%, 47.15% ± 5.15%, and 31.99% ± 5.49% for TCPS and 85.66% ± 4.84%, 49.67% ± 3.46%, and 39.53% ± 3.45% for collagen IV‐coated TCPS, respectively, when compared to unmodified ADSCs. However, adhered cells displayed normal cell spreading morphology (Figure 2f; Figure S4c, Supporting Information). Taken together, these results suggested that DPP modification did not significantly alter essential ADSC functionality. We subsequently investigated whether the reduced adherence to substrate surfaces (TCPS and collagen IV‐coated TCPS) affected P‐selectin‐mediated binding.

### 2.4 In Vitro and In Vivo Targeting Properties of DPP‐ADSCs

**Figure 3:**
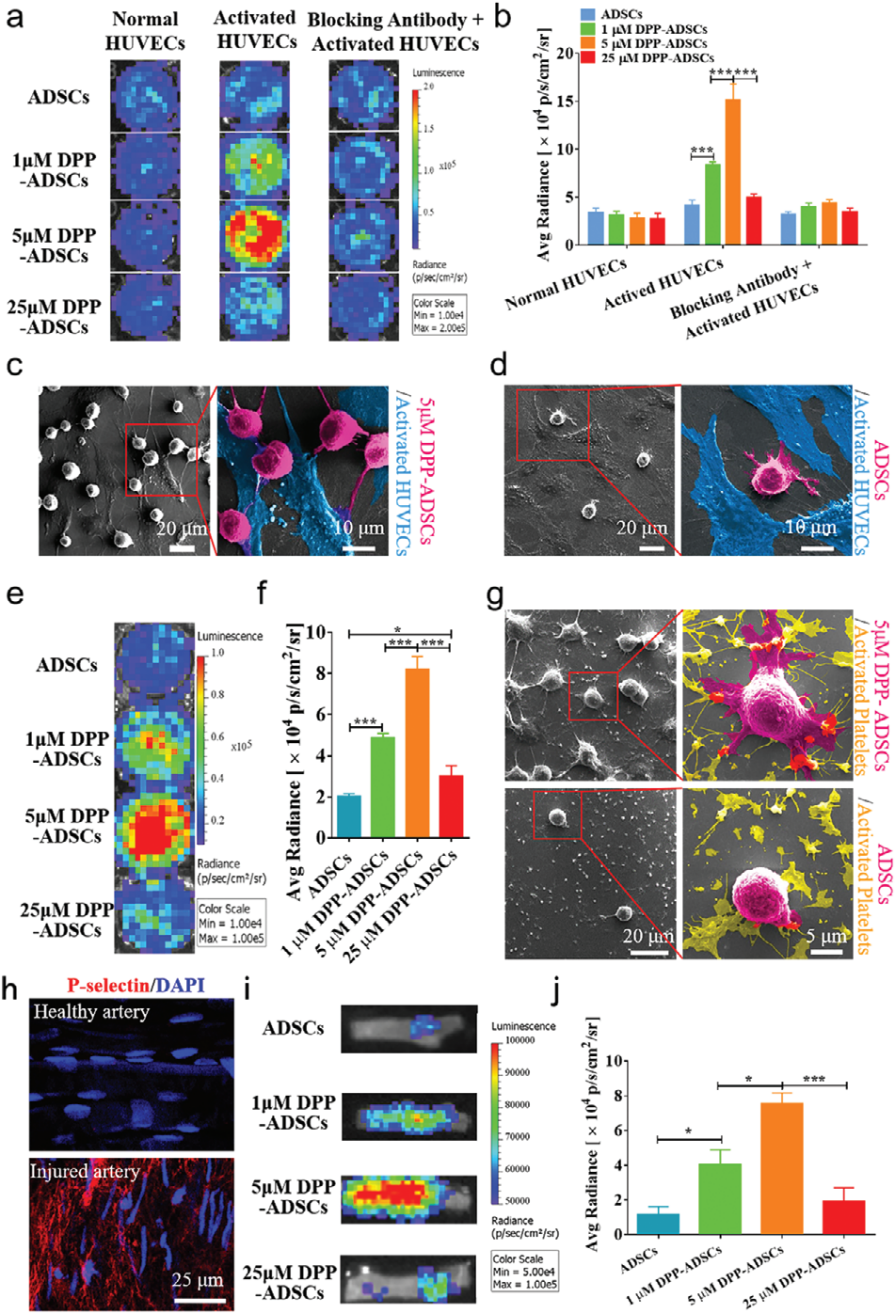
The in vitro*/*in vivo targeting properties of DPP‐ADSCs. a) After shaking incubation for 1 h, binding of ADSCs with or without DPP modification to normal, TNF‐*α* activated HUVECs, or TNF‐*α* activated HUVECs incubated with P‐selectin blocking antibody was observed by BLI (*n* = 5) and b) quantitatively analyzed based on Luc fluorescence intensities. c,d) SEM images of ADSCs and 5 × 10^−6^ m DPP‐ADSCs (magenta cells) binding to TNF‐*α*‐activated HUVECs (blue cells). e) After shaking incubation for 1 h, the targeted binding of DPP modified and unmodified ADSCs binding to ADP activated platelets was observed by BLI (*n* = 5) and f) quantitatively analyzed based on Luc fluorescence intensities. g) SEM images of ADSCs and 5 × 10^−6^ m DPP‐ADSCs (magenta cells) binding with ADP‐activated platelets (yellow cells). h) En face staining of P‐selectin on the lumen surface of rat healthy and injured femoral artery. i) At 10 min after injection of cells, the targeted binding of DPP‐modified and unmodified ADSCs binding to healthy and injured femoral artery was observed by BLI (*n* = 4) and j) quantitatively analyzed based on Luc fluorescence intensities.

After modification with 1 × 10^−6^, 5 × 10^−6^, and 25 × 10^−6^ m DPP for 10 min, firefly luciferase‐transfected ADSCs (Luc‐ADSCs) were utilized to assess in vitro targeted binding. To mimic P‐selectin overexpression at vascular injury sites, human umbilical vein endothelial cells (HUVECs) and platelets were activated by pretreating with tumor necrosis factor‐*α* (TNF‐*α*) and adenosine diphosphate (ADP), respectively. Immunofluorescence staining showed that activated HUVECs displayed more P‐selectin compared to normal HUVECs (Figure S5, Supporting Information). DPP‐modified and unmodified Luc‐ADSCs showed weak binding to normal HUVECs; bioluminescence imaging (BLI) signals remained low and comparable among inactivated groups (**Figure 3**a,b). However, Luc‐ADSCs modified with different concentrations of DPP showed varying binding potentials to activated HUVECs (Figure 3b). Quantification of BLI data showed that 5 × 10^−6^ m DPP‐Luc‐ADSCs possessed the strongest binding to activated HUVECs, which was approximately twofold higher than 1 × 10^−6^ m DPP‐Luc‐ADSCs group and fourfold higher than unmodified and 25 × 10^−6^ m DPP‐Luc‐ADSCs groups (Figure 3b). P‐selectin blocking antibody inhibited the targeted binding of DPP‐ADSCs to activated HUVECs, as evidenced by the BLI intensity of the DPP‐ADSCs group falling to levels comparable to normal HUVEC cultures (Figure 3a,b). These data indicated that PBP was the principal mediator of ADSC binding to P‐selectin. Binding of DPP‐Luc‐ADSCs to activated platelets was also analyzed, and 5 × 10^−6^ m DPP‐Luc‐ADSCs also showed the strongest binding capacity to activated platelets (Figure 3e,f). Low‐magnification scanning electron microscope (SEM) images showed that 5 × 10^−6^ m DPP‐ADSCs bound more effectively than unmodified ADSCs, to activated HUVECs and platelets (Figure 3c,d,g). Quantitative analyses, based on low‐magnification SEM images, indicated that the number of 5 × 10^−6^ m DPP‐ADSCs bound to activated HUVECs and platelets was approximately fivefold to sixfold greater than the number of unmodified ADSCs (Figure S6, Supporting Information). High‐magnification SEM images clearly showed the different adhesion states of modified and unmodified ADSCs (Figure 3c,d,g). The 5 × 10^−6^ m DPP‐ADSCs were more inclined to bind with activated HUVECs, while the adhesion of unmodified ADSCs was random. Some unmodified ADSCs adhered to the space among the activated HUVECs. The 5 × 10^−6^ m DPP‐ADSCs spread more on activated platelet layers than the unmodified ADSCs, and the majority of cellular pseudopodia were observed to be bound to activated platelets.

Wire‐mediated rat femoral artery injury (Figure S7, Supporting Information) induced luminal expression of P‐selectin within 10 min (Figure 3h). To assess DPP‐ADSCs binding in vivo, injured vessels were examined by BLI 10 min after injection of 1 × 10^−6^, 5 × 10^−6^, and 25 × 10^−6^ m DPP‐Luc‐ADSCs. As with in vitro results, 5 × 10^−6^ m DPP‐Luc‐ADSCs showed the strongest targeting capacity to P‐selectin‐rich injury sites (Figure 3i,j). Injured vessels were almost completely covered by intense BLI signal in the 5 × 10^−6^ m DPP‐Luc‐ADSCs (Figure S8, Supporting Information). These data suggested that 5 × 10^−6^ m DPP‐Luc‐ADSCs had superior binding capacity to activated ECs and platelets at arterial injury sites.

### 2.5 Repair of Arterial Injury by DPP‐ADSCs

**Figure 4:**
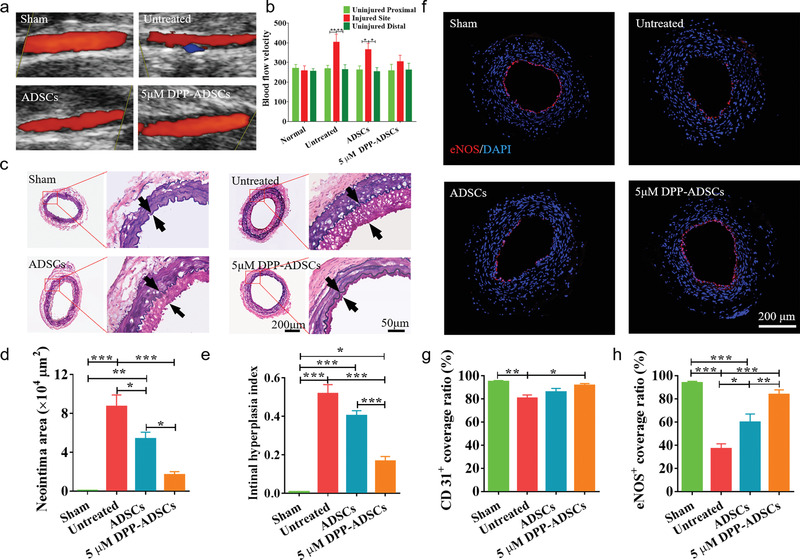
The effect of DPP‐ADSCs treatment on reparation of injured arteries after 21 days. The a) patency and b) blood flow of injured arteries in sham, untreated, ADSCs injection. and 5 × 10^−6^ m DPP‐ADSCs injection groups was measured by color Doppler ultrasound. c) VVG staining of cross sections of injured arteries with different treatment. The d) neointima area and e) intimal hyperplasia index were calculated based on the VVG staining to reflect the histopathological characteristics of injured arteries in the different treatment groups. f) Refunctional endothelialization of injured arteries was analyzed by immunofluorescence staining with anti‐eNOS. The quantitative analysis of g) CD31‐positive and h) eNOS‐positive endothelial coverage rate based on merge images at 21 days. All data are representative of (*n* = 5) animals per group.

Based on the superior in vitro targeted binding to activated HUVECs (Figure 3a,b) and platelets (Figure 3e,f), and in vivo targeted binding to injured vessels (Figure 3i,j), 5 × 10^−6^ m DPP‐ADSCs were selected for administration by systemic injection into wire‐mediated rat femoral artery injury models, for subsequent evaluation of the reparative effects. After 21 days, color Doppler ultrasound (**Figure** **4**a) and stereomicroscopy (Figure S9, Supporting Information) images showed that all vessels had patency, except one that was obstructed in the untreated group. The blood flow velocity of injured vessels in the 5 × 10^−6^ m DPP‐ADSCs treatment group was slightly higher than in the proximal and distal healthy vessels, adjacent to the injured site. In contrast, blood flow velocity of injured vessels in untreated and unmodified ADSCs groups was significantly higher than in proximal and distal healthy vessels (Figure 4b). Narrowness of injured vessels was reflected in hemodynamic results and was consistent with luminal diameters measured by color Doppler ultrasound (Figure 4a,b). The reparative effect of 5 × 10^−6^ m DPP‐ADSCs was evaluated by histological analysis. Elastin staining was used to identify internal and external elastic lamina and evaluate neointimal hyperplasia occurrence (Figure 4c). At 21 days, extensive neointima hyperplasia was observed in the untreated group. ADSC treatment significantly reduced neointima hyperplasia, compared with untreated group (*p* = 0.034), but this was not as potent as 5 × 10^−6^ m DPP‐ADSCs treatment (*p* < 0.001) (Figure 4d). The intimal hyperplasia index of 5 × 10^−6^ m DPP‐ADSCs group was 0.17 ± 0.05, which was considerably less than 0.52 ± 0.09 (*p *< 0.001) of untreated and 0.40 ± 0.05 (*p* < 0.001) of unmodified ADSCs treatment groups (Figure 4e).

Ideal methods for repair of injured arteries should aim to recover the original physiological structure. Therefore, we first used anti‐CD31 immunofluorescence staining to examine endothelium regeneration at 21 days post‐surgery (Figure S10, Supporting Information). Results revealed that the 5 × 10^−6^ m DPP‐ADSCs treatment group had arterial lumens almost fully covered by CD31^+^ ECs, similar to natural healthy arterial endothelium. Quantification showed that coverage rates of the CD31^+^ ECs in all groups were over 80%, and the 5 × 10^−6^ m DPP‐ADSCs group had reached over 90% (Figure 4g). In addition, endothelial NO synthase‐positive (eNOS^+^) cells were visualized to assess the recovery of functional endothelium (Figure 4f). Figure 4h indicated that the coverage rate of eNOS^+^ ECs in the 5 × 10^−6^ m DPP‐ADSCs group was 83.91% ± 8.81%, which was significantly higher than that of untreated (37.03% ± 9.63%, *p *< 0.001) and unmodified ADSCs groups (59.76% ± 16.15%, *p* = 0.008).

After 21 days, VSMC alignment in injured vessels was detected by anti‐*α*‐smooth muscle actin (*α*‐SMA) immunostaining (Figure S11d, Supporting Information). VSMCs were circumferentially aligned in healthy arteries (sham group) but had a disordered arrangement in untreated injured arteries. ADSCs treatment had a modest effect on recovering VSMCs circumferential alignment, whereas treatment with 5 × 10^−6^ m DPP‐ADSCs enhanced alignment recovery; only the innermost layer of VSMCs had absence of orientation (Figure S11d, Supporting Information). The aligned cell nuclei rate and the nuclear shape index of *α*‐SMA^+^ VSMCs following 5 × 10^−6^ m DPP‐ADSCs treatment were lower than that in healthy arteries (sham group), but were approximately threefold higher than those of untreated group and approximately twofold higher than those of unmodified ADSC‐only treatment group (Figure S11e,f, Supporting Information).

### 2.6 Mechanisms of Repair by DPP‐ADSCs

Whether ADSCs differentiated after targeting to vascular injury sites was evaluated by injecting 5 × 10^−6^ m DPP‐modified and unmodified green fluorescent protein positive (GFP^+^) ADSCs at 21 days. ADSCs were harvested from rats ubiquitously expressing GFP, and FCM and inverted fluorescence microscopy confirmed that the majority of ADSCs were GFP‐positive (Figure S11a,b Supporting Information). Results revealed that ADSCs had not differentiated into ECs (co‐staining with anti‐GFP and anti‐CD31, Figure S11c, Supporting Information) or VSMCs (co‐staining with anti‐GFP and anti‐*α*‐SMA, Figure S11d, Supporting Information), regardless of the addition of DPP modification. Subsequently, cell retention time at vascular injury sites was assessed (Figure S11g, Supporting Information). Confocal images showed that the retention time of 5 × 10^−6^ m DPP‐GFP^+^ADSCs was at least 7 days, while GFP signal was undetectable at 7 days when unmodified GFP^+^ADSCs were administered. At 14 and 21 days, both groups had no observable GFP^+^ADSCs at vascular injury sites. The number of 5 × 10^−6^ m DPP‐GFP^+^ADSCs was more than in unmodified GFP^+^ADSCs at 1, 4, and 7 days; but there was a correlative decrease in the numbers of GFP^+^ADSCs binding to the lumen of injured vessels with increasing post‐operation time in both groups.

**Figure 5:**
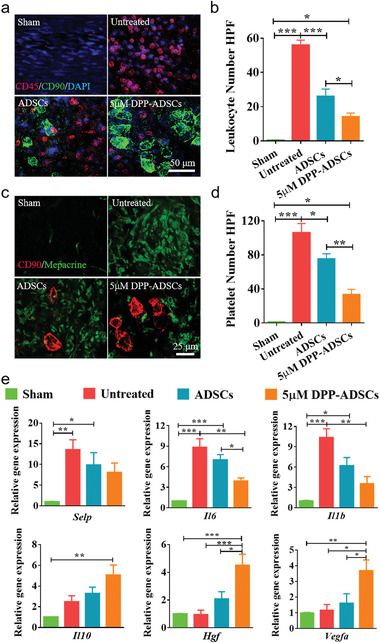
Shielding and inflammatory regulation effects of DPP‐ADSCs targeted to injured arteries at 24 h post‐surgery. a) The leukocyte adhesion shielding effect of ADSCs bound to injured vessels was observed by en face immunostaining using anti‐CD45 and anti‐CD90. b) The adhesion number of leukocytes was calculated based on en face staining. c) The platelet adhesion‐shielding effect of ADSCs bound to injured vessels was observed by en face immunostaining using mepacrine and CD90. d) The adhesion number of platelets was calculated based on en face staining. e) The relative expression of inflammatory genes (*Selp*, *Il6*, *Il1b*, and *Il10*) and pro‐regeneration genes (*Hgf* and *Vegfa*) of injured arteries across the different treatments. All data are representative of (*n* = 5) animals per group.

Platelet and leukocyte adhesion to injured arteries was observed at 24 h post‐injury. Co‐staining images showed that, compared with other groups, 5 × 10^−6^ m DPP‐ADSCs treatment resulted in coverage with more ADSCs at the lumen of the injured arteries (**Figure 5**a,c). ADSC coverage rate (ADSC‐positive area/whole field area) was calculated based on the images co‐stained with CD45 and CD90. Results showed that 5 × 10^−6^ m DPP‐ADSCs had significantly higher coverage rates (25.19% ± 8.21%), compared to unmodified ADSCs (3.81% ± 3.43%, *p* = 0.0013). Injury site coverage by 5 × 10^−6^ m DPP‐ADSCs effectively reduced leukocyte (Figure 5b) and platelet (Figure 5d) adhesion, compared to untreated and ADSCs treatment groups. Our in vitro results confirmed this shielding effect by 5 × 10^−6^ m DPP‐ADSCs (Figure S12, Supporting Information). Inflammatory genes were significantly reduced (*Selp*, *Il6*, *Il1b*, and *Il10*) and there was increased expression in pro‐regeneration genes (*Hgf*, *Vegfa*), when injured vessels were treated with 5 × 10^−6^ m DPP‐ADSCs (Figure 5e).

**Figure 6:**
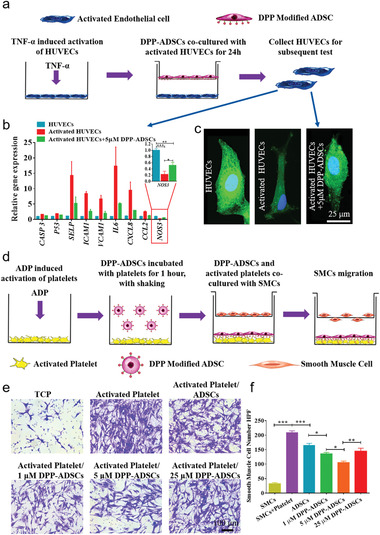
The regulatory effects of DPP‐ADSCs on activated HUVECs and platelets. a) Schematic illustration of the coculture model used. The b) representative gene expression analysis (*n* = 5) and the c) intracellular NO level testing of activated HUVECs after coculture for 24 h (*n* = 3). The normal HUVECs and activated HUVECs without cocultured 5 × 10^−6^ m DPP‐ADSCs were used as positive and negative control groups, respectively. d) Schematic illustration of chemotaxis experiment for analyzing the regulatory effect of ADSCs on VSMCs migration caused by activated platelets. e) Representative images of the transwell migration assay of VSMCs after 12 h of coculture with activated platelets, as regulated by ADSCs in different conditions (*n* = 3). f) Quantitative analysis of the migrated VSMCs per microscopic field view across the different groups.

Following TNF‐*α* activation, HUVECs were cocultured with 5 × 10^−6^ m DPP‐ADSCs for 24 h (**Figure** **6**a). The qRT‐PCR results showed that 5 × 10^−6^ m DPP‐ADSCs significantly decreased apoptosis (*CASP3*, *P53*) and inflammatory (*SELP*, *ICAM1*, *VCAM1*, *IL6*, *CXCL8*, *CCL2*) gene expression, while recovering functional (*NOS3*) gene expression in activated HUVECs (Figure 6b). 3‐Amino,4‐aminomethyl‐2ʹ,7ʹ‐difluorescein diacetate (DAF‐FM) fluorescence tests indicated that cocultured ADSCs recovered intracellular nitric oxide (NO) in activated HUVECs (Figure 6c). We also found that ADSCs inhibited chemotaxis of VSMCs toward activated platelets (Figure 6d–f). Inhibitory effects were dependent on numbers of ADSCs bound to activated platelets (Figure 3e,f). Therefore, inhibition was strongest in the 5 × 10^−6^ m DPP‐ADSCs group (Figure 6f).

### 2.7 The Targeting Ability of DPP‐ADSCs to Balloon‐Injured Human Femoral Arteries

**Figure 7:**
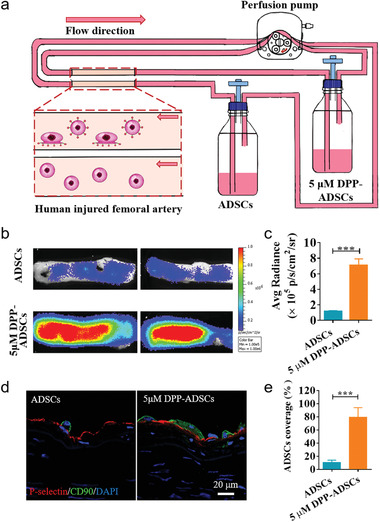
The targeting properties of DPP‐hADSCs to balloon‐injured human femoral arteries. a) Schematic illustration of hADSCs dynamic targeting experiment. b) After dynamic culture for 1 h, 5 × 10^−6^ m DPP‐modified and unmodified hADSCs binding to injured human femoral arteries were observed by BLI (*n* = 4) and c) quantitatively analyzed based on Luc fluorescence intensities. d) The P‐selectin expression and adhesion of hADSCs on the lumen surface of injured arteries in different groups was observed by co‐staining using anti‐P‐selectin and anti‐CD45 antibodies (*n* = 3). e) Quantitative analysis of the adhesion of hADSCs per microscopic field from the 5 × 10^−6^ m DPP‐modified and unmodified hADSCs groups.

Angioplasty‐induced human femoral arteries were placed in a dynamic flow culture system, as shown in **Figure** **7**a and Figure S13a, Supporting Information. After 1 h of flow culture, BLI showed that targeted binding of human‐ADSCs(hADSCs) modified with 5 × 10^−6^ m DPP to injured human femoral arteries produced a significantly stronger signal than that of unmodified hADSCs (*p* < 0.001) (Figure 7b,c). Both en face staining (Figure S13b, Supporting Information) and immunofluorescence imaging (Figure 7d) indicated that P‐selectin overexpression was observable on the luminal surface of injured arteries. Co‐staining images showed that the number of CD90^+^ ADSCs bound to P‐selectin overexpressing injured arteries in the 5 × 10^−6^ m DPP‐ADSCs group was greater than that in the unmodified ADSCs group (Figure 7d). Quantification of ADSCs coverage rate was calculated as 79.30% ± 13.80% in the 5 × 10^−6^ m DPP‐ADSCs group, which was approximately fivefold greater than that in the unmodified ADSCs group (Figure 7e). Therefore, these data confirmed that 5 × 10^−6^ m DPP modification improved ADSCs targeting to injured human vessels.

## 3 Discussion

**Figure 8:**
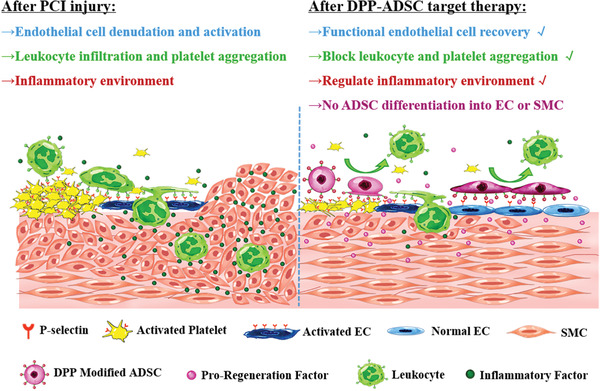
Mechanism illustration of 5 × 10^−6^ m DPP‐ADSCs target therapy for vascular injury.

Development of effective and safe methods for the prevention and treatment of PCI‐related complications (thrombosis, endothelium denudation, inflammation, intimal hyperplasia) presents a difficult scientific challenge to circumvent. Cellular therapy by systematic injection has shown some promising results in repairing vascular injuries.^[^ ^32^ ^]^ However, treatment efficacy remains elusive due to poor targeting and retention of cells at injured vasculature.^[^ ^33^ ^]^ Clinical studies and animal experiments confirm that optimization of cellular targeting improves cell therapy.^[^ ^34^ ^]^ A variety of factors decrease homing of cells to injured sites; one typical influencing factor is the absence of relevant cell‐surface targeting ligands.^[^ ^19a^ ^]^ Therefore, endowment of cells with appropriate targeting ligands can enhance cell capacity to specifically bind to vascular injury sites. We aimed to target the rapid upregulation of P‐selectin on surfaces of injured vessels,^[^ ^5^ ^]^ by addition of PBP‐displaying DMPE‐PEG linkers into the external cell membrane of ADSCs. DPP‐ADSCs had remarkable targeting capabilities to injured arteries and provided pro‐regenerative therapeutic effects (summarized in **Figure** **8**).

Using the approach reported here, ADSC modification was achieved within 10 min (Figure 1c,e) through mixing of premade DPP with ADSCs. This approach was without risk of decreased viability, proliferation, migration, or paracrine secretion (Figure 2a–c; Figure S3, Supporting Information). Thus, DMPE‐PEG‐based cell modification approaches may offer clinically viable strategies to enhance cell treatment of vascular injuries. Furthermore, human relevance and clinical accessibility was demonstrated by the in vitro dynamic flow culture system used in this investigation (Figure 7), and significantly higher hADSCs coverage rates were observed in the injury sites of human femoral arteries.

PEG is an inert polymer widely used to modify cells due to its inhibition of protein adsorption,^[^ ^24b^ ^]^ nonspecific cellular adhesion,^[^ ^35^ ^]^ and its improvements to cell circulation time.^[^ ^36^ ^]^ Previously, ligand‐conjugated PEG lipids were inserted into extracellular vehicle (EV) membranes, which reduced nonspecific interactions and prolonged circulation times, showing potential in promoting EV accumulation at target tissue sites.^[^ ^37^ ^]^ The use of PEG in our DPP molecule may have contributed to the reduced ADSCs adhesion to TCPS (Figure 2d–f) and collagen IV‐coated TCPS (Figure S4, Supporting Information); a similar result was reported by Iwata and co‐workers.^[^ ^38^ ^]^ This observation can be explained by excessive PEG modification resulting in a strong inhibition of other membrane‐bound proteins binding their ligands, significantly reducing the potential of the targeted delivery systems.^[^ ^39^ ^]^ Our results indicated that the balance between anti‐nonspecific adhesion and targeted binding ability should form an important consideration when modifying cell surfaces. High concentrations (25 × 10^−6^ m) of DPP resulted in cells being more prone to show PEG anti‐adhesion characteristics, rather than the targeting ability of PBP. Therefore, targeted binding to activated platelets and HUVECs by 25 × 10^−6^ m DPP‐ADSCs was significantly lower than that of 5 × 10^−6^ m DPP‐ADSCs (Figure 3a–g). In vivo experiments confirmed this finding, as 5 × 10^−6^ m DPP‐ADSCs had the strongest targeting ability to injured vessels (Figure 3i,j), indicating an appropriate balance between PEG inhibition of nonspecific binding and the DPP‐specific binding to P‐selectin was achieved.

Activated platelet adhesion to injured endothelium triggers a release of proinflammatory factors to induce a state of vascular inflammation.^[^ ^40^ ^]^ Further release of proinflammatory factors, such as IL‐1 and IL‐6, is secreted by both leukocytes and proinflammatory ECs,^[^ ^9^ ^]^ exacerbating inflammation.^[^ ^41^ ^]^ Cumulatively, these factors induce proliferation and migration of VSMCs,^[^ ^10^ ^]^ which ultimately leads to intimal hyperplasia. Previous studies have shown that competitive binding of nanoparticles to injured vascular walls suppressed accumulation of platelet adhesion and platelet‐mediated reactions, sequentially decreasing neointima formation.^[^ ^4b^ ^]^ Similarly, in our study, DPP‐ADSCs binding and spreading on the surface of the injured vessels occupied P‐selectin binding sites, blocking platelets and leukocyte adhesion at the vascular injury sites (Figure 5a–d). The immunoregulatory function of ADSCs may have also played a role in the observed dampening of inflammation. After 24 h of treatment, we detected high expression of immunomodulatory IL‐10 at the site of injury, and the expression of proinflammatory IL‐1 and IL‐6 was reduced (Figure 5e). IL‐10 secreted by ADSCs serves as an important immunosuppressing cytokine in cardiovascular disease,^[^ ^42^ ^]^ decreasing proinflammatory chemokine production and alleviating the inflammatory microenvironment.^[^ ^43^ ^]^ Therefore, targeted delivery of DPP‐ADSCs had a synergistic immunomodulatory effect; the reduction of platelet and leukocyte adhesion accompanied by ADSC paracrine secretion of anti‐inflammatory factors.

The regeneration of mature endothelium is essential for the maintenance of vascular patency and the inhibition of intimal hyperplasia. Therefore, re‐endothelialization was used as a key indicator to evaluate therapeutic effects. Riegler et al. used superparamagnetic iron oxide nanoparticles to target the delivery of MSCs for vascular injury treatment.^[^ ^4a^ ^]^ Their magnetic targeting system reduced restenosis after 3 weeks, and the coverage of CD31^+^ECs was over 80% of the lumen surface. Similarly, in our work, we observed almost complete coverage by CD31^+^ ECs at the injury site after treatment using both ADSCs and DPP‐ADSCs (Figure 4g; Figure S10, Supporting Information), whereas there was a significant difference in the intimal hyperplasia index (Figure 4e) and functional ECs coverage (Figure 4f,h) between the two groups. eNOS is an essential component of functional vasculature and homeostasis.^[^ ^44^ ^]^ Our results showed that DPP‐ADSCs treatment increased functional endothelium coverage, as assessed at 21 days (Figure 4f,h). This may be ascribed to ADSCs paracrine secretion of a multitude of bioactive factors that improved endothelial regeneration. For example, hepatocyte growth factor (HGF) remodels damaged ECs intercellular junctions and reduces permeability,^[^ ^45^ ^]^ and vascular endothelial growth factor (VEGF) promotes ECs proliferation.^[^ ^46^ ^]^ Our in vitro experiments demonstrated that ADSCs could effectively recover endothelial dysfunction and restore NO synthesis ability (Figure 6a–c). Heightened gene expression of HGF and VEGF was also detected in vivo after DPP‐ADSCs treatment (Figure 5e), which were predictive of enhanced regeneration.

VSMCs phenotypic switching plays important roles in vascular remodeling and disease development.^[^ ^47^ ^]^ VSMCs morphology has been reported to be linked to phenotype and function.^[^ ^48^ ^]^ In adult mammalian tunica media, VSMCs exhibit a typically elongated spindle shape with contractile ability and low rate of proliferation. VSMCs in a disease state have proliferative phenotypes with hypertrophic polygonal morphology.^[^ ^49^ ^]^ After PCI, loss of ECs signaling and subsequent intense inflammatory responses stimulate VSMCs phenotypic switching, leading to changes in morphology and invasiveness,^[^ ^50^ ^]^ which conformed with our results (Figure S11d, Supporting Information). Following treatment with DPP‐ADSCs, VSMCs at the injured site were oriented in a radial array manner and had cellular morphologies that maintained a close resemblance to VSMCs in healthy vessels. This result was related to the enhanced functional endothelium regeneration and the attenuated inflammatory response by DPP‐ADSCs treatment. Interestingly, our in vitro experiments showed that ADSCs had the regulatory ability to inhibit activated platelet‐induced VSMCs migration (Figure 6d–f). Specific mechanisms of this interaction still require further clarification in future investigations. The lowered inflammatory response and improved functional endothelium regeneration at the injury site could prevent excessive proliferation of VSMCs and help to reduce the incidence of intimal hyperplasia. This was evidenced by the Doppler ultrasound results (Figure 4a,b) and the statistical analysis of intimal hyperplasia index (Figure 4c–e).

DPP‐modified living cells may have broader application in the treatment of cardiovascular diseases and related conditions. Post‐ischemic renal tissues^[^ ^51^ ^]^ have elevated expression of P‐selectin, and we also identified a similar increase in myocardial infarction areas (Figure S14a, Supporting Information). Therefore, DPP stem cells could potentially be used to enhance therapeutic efficacy of stem cell therapy for these diseases. The lumen surface of artificial vascular implants (e.g., vascular grafts; Figure S14b, Supporting Information), intravascular stents,^[^ ^52^ ^]^ and cardiac valves^[^ ^53^ ^]^ also exhibits elevated expression of P‐selectin post‐implantation. Preseeding of ECs onto implants has achieved promising laboratorial results in rapid re‐endothelialization. However, long cell culture times and seeding periods, EC source, and desquamation of seeded ECs remain obstacles for the further development of this method.^[^ ^54^ ^]^ Induced pluripotent stem cell‐derived ECs (iPSCs‐ECs) have recently emerged as an attractive cell type for replacing ECs due to advantages associated with being patient‐specific (eliminating the need for immunosuppression) and having an abundant cell source for iPSCs generation (offering an unlimited source for ECs).^[^ ^55^ ^]^ Thus, we propose that systemic injection of DPP‐modified iPSCs‐ECs may offer an alternative method for the promotion of rapid endothelialization of cardiovascular implants—a direction that warrants future investigation.

## 4 Conclusion

In this study, we successfully modified PBP on the surface of living ADSCs using DMPE‐PEG and amply confirmed that ADSCs modified with 5 × 10^−6^ m DPP for 10 min possessed remarkably stronger targeted binding to activated platelets, activated HUVECs, and injured vessels. The intra‐arterial injection of 5 × 10^−6^ m DPP‐ADSCs significantly improved re‐endothelialization, maintained the circumferential alignment of VSMCs, and decreased neointimal hyperplasia of injured vessel compared to unmodified ADSCs. Importantly, this approach is rapid and convenient, and has no influence on viability, apoptosis, and paracrine secretion of cells, which provided a clinically receivable platform for delivering reparative cells to P‐selectin high‐expressed sites via system injection.

## 5 Experimental Section

##### Materials

PBPs were custom synthesized by GL Biochem (Shanghai, China) with purity >99%. DMPE‐PEG‐MAL (MW 5 kDa) was purchased from Ponsure Biotechnology (Shanghai, China). Human platelet‐rich plasma (PRP) was purchased from the Tianjin Blood Center (Tianjin, China). HUVECs and endothelial cell medium (ECM) were purchased from ScienCell (California, USA). hADSCs were purchased from Cyagen Biosciences (Suzhou, China). Lipofectamine 3000 transfection reagent was purchased from Invitrogen (Life Technologies, California, USA). Plasmid overexpressing firefly luciferase was purchased from MiaoLing Plasmid (Wuhan, China). D‐Luciferin potassium salt was obtained from Gold Biotechnology (Buffalo, USA). The detailed information about other materials used in this study is available in the Supporting Information.

##### Animal Use and Study Approval

Sprague‐Dawley (SD) rats, GFP^+^ SD rats (male, aged 8 weeks and weight 250–280 g), were obtained from the Laboratory Animal Center of the Academy of Military Medical Sciences (Beijing, China). All animal experiments were approved by the Animal Experiments Ethical Committee of Nankai University and were in accordance with the NIH Guide for Care and Use of Laboratory Animals. The accreditation number of the laboratory is SYXK(Jin) 2019‐0003 promulgated by Tianjin Science and Technology Commission.

##### Preparation of DPP and DPP‐FITC

A cysteine (C) was added to the N‐terminus of PBP (DAEWVDVS) to produce a thiol on the N‐terminus, which can react with MAL. Thus, DMPE‐PEG‐MAL was used for preparation of DPP conjugates. The detailed procedure of DPP synthesis is available in the Supporting Information. The chemical structure of DPP was characterized by NMR (^1^H NMR, Mercury Vx‐300), using D_2_O as the solvent.

DPP‐FITC was synthesized by adding an excess amount of FITC (10‐fold molar excess) to DPP aqueous solution with stirring under dark condition. After 24 h, the mixture was dialyzed against deionized water with dialysis membrane for 3 days, and then lyophilized to obtain the DPP‐FITC powder.

##### Rat‐ADSCs Isolation, Cultivation, Identification, and Transfection

Rat‐ADSCs were isolated, cultured, and identified according to previous report.^[^ ^56^ ^]^ The detailed procedure is available in the Supporting Information. The fourth passage cells were used for subsequent experiments.

For the firefly luciferase transfection, rat ADSCs were seeded in six‐well flat‐bottomed micro‐assay plates at a density of 10^5^ cells per well. At 70–80% confluence, cells were transfected with 2 µg of plasmid carrying the firefly luciferase reporter gene using Lipofectamine 3000. After incubation for 48 h, firefly luciferase‐transfected rat ADSCs (Luc‐rat ADSCs) were collected for subsequent experiments.

##### ADSCs Modification

The rat ADSCs were harvested by centrifugation (1000 rpm, 5 min, room temperature (RT)) after tryptic digestion. The harvested cells were washed twice with phosphate buffer saline (PBS) prior to use in experiments.

To optimize the modification concentration of DPP, 5 × 10^5^ rat ADSCs were resuspended in 500 µL of serum‐free DMEM/F12 medium with different DPP‐FITC concentrations. After 10 min of incubation at RT under gentle agitation, the cells were washed with PBS by centrifugation (1000 rpm, 5 min, RT). The FITC fluorescence signal was immediately analyzed using a flow cytometer and a confocal laser scanning microscope. The optimal concentration was determined when the fluorescent intensity reached saturation. FITC‐only was used as a control group and the fluorescence signal was analyzed using the same protocol.

To optimize the modification time of DPP, 5 × 10^5^ rat ADSCs were resuspended in 500 µL of serum‐free DMEM/F12 medium with 5 × 10^−6^ and 25 × 10^−6^ m DPP‐FITC. After incubation for different times at RT under gentle shaking, the cells were washed with PBS by centrifugation (1000 rpm, 5 min, RT). The FITC fluorescence signal was tested as described above. The optimal time was determined when the fluorescent intensity reached saturation.

To evaluate the modification kinetics, rat ADSCs modified with 25 × 10^−6^ m DPP‐FITC for 10 min were incubated at 37 °C DMEM/F12 medium with or without 10% FBS for up to 4 h. The FITC fluorescent intensity was tested using a flow cytometer at different time points. The MFI of as‐modified rat ADSCs was defined as 100% MFI. The RFI of rat ADSCs incubated at 0.5, 1, 2, and 4 h were counted to evaluate the modification kinetics. After incubation for 4h, the FITC fluorescent signal was observed using a confocal laser scanning microscope.

##### Targeted Binding of DPP‐ADSCs to Activated HUVECs and Platelets

Glass slides with appropriate size were placed in 48‐well polystyrene plates (*n* = 5). HUVECs were cultured on glass slides and activated by TNF‐*α* (described in Supporting Information) followed by incubation in ECM culture medium with or without P‐selectin neutralizing antibody (10 µg mL^−1^) for 20 min. After rinsing three times with fresh ECM culture medium, 500 µL of unmodified or modified Luc‐rat ADSCs suspension (4 × 10^4^ cells mL^−1^) was added to per well and shaking incubated at 37 °C. One hour later, the unbound cells were removed by rinsing three times with PBS and 100 µL D‐Luciferin solution (150 µg mL^−1^ in PBS) was added. The signal of Luc‐rat ADSCs was collected by in vivo Imaging System IVIS Luminar to test the targeted binding of DPP‐rat ADSCs to activated HUVECs. The normal HUVECs as control group were used to analyze target property of DPP‐rat ADSCs with the same protocol.

The targeted binding of DPP‐rat ADSCs to activated platelet was tested by the same method with activated HUVECs. A total of 450 µL human PRP was added to each well, and subsequently 50 µL 0.5 mmol ADP solution was added to activate platelets. After incubation at 37 °C for 1 h, the inactivated platelets were removed by washing three times with PBS. Then the activated platelets were used for target analysis.

In order to observe morphology and to calculate the number of unmodified and 5 × 10^−6^ m DPP‐rat ADSCs bound to activated HUVECs or platelets, the glass slides were fixed with 2.5% glutaraldehyde, dehydrated with serial dilutions of ethanol, and examined by SEM. Three images per sample and five samples per group were used to calculate the bound cell numbers.

##### In Vivo Targeted Binding of DPP‐ADSCs to Rats Injured Femoral Arteries

A wire‐mediated vascular injury was induced in the SD rat femoral artery (details were described in Supporting Information). After being injured for 10 min, rats randomly received 200 µL of DPP‐modified or unmodified Luc‐rat ADSCs suspension (1 × 10^7^ mL^−1^ in 5 UI mL^−1^ heparin) by injection at the site of femoral artery proximal to the inguinal ligament with a 30 gauge needle (BD). This cell delivery approach simulates the clinically used catheter‐based cell delivery method, which is performed extensively and easily integrated into PCI procedures.^[^ ^57^ ^]^ After injection for 10 min, the injured femoral arteries were harvested and placed into 24‐well plates. A total of 200 µL D‐Luciferin solution (150 µg mL^−1^ in PBS) was added. The BLI signal of Luc‐rat ADSCs was tested to evaluate the target potential of DPP‐rat ADSCs to vascular injured site.

##### The Reparative Effect of DPP‐ADSCs on Vascular Injury

After wire‐mediated femoral arteries injury, SD rats were randomly selected to receive 2 × 10^6^ rat ADSCs (ADSCs group), 5 × 10^−6^ m DPP‐rat ADSCs (5 × 10^−6^ m DPP‐ADSCs group), or saline (untreated group). Treatments were administered in a volume of 200 µL saline solution containing 5 UI mL^−1^ heparin. Injection was performed as described above. Afterwards, the skin incision was closed with surgical sutures. Rats in sham group just only received femoral arteries separation without wire‐mediated injury and any other treatment. At 24 h after the operation, the femoral arteries (*n* = 5 of each group) were taken out and cut into two parts from the middle. One part was used to test gene expression by real‐time PCR. The other part was cut into two equally parts along the axial direction. One section was used to evaluate coverage of ADSCs on injured vessels and their effect on shielding leukocytes adhesion by en face co‐immunostaining with anti‐CD45‐PE (BioLegend, 202207) and anti‐CD90‐FITC (BioLegend, 202503). The other section was used to assess ADSC binding effects on shielding platelet adhesion, by en face co‐staining with mepacrine and anti‐CD90‐PE (BioLegend, 202523). At 21 days, the patency and blood velocity of injured femoral arteries (*n* = 5 of each group) were first visualized by high‐resolution ultrasound (Vevo 2100 System, Canada) after the rats were anesthetized with isoflurane. Then rats were sacrificed by injection of an overdose of pentobarbital sodium. The injured femoral arteries were photographed, fixed with 4% paraformaldehyde, and embedded in optimal cutting temperature (OCT) compound (Tissue‐Tek) for frozen cross sections. The sections were stained by Verhoeff–Van Gieson (VVG) to assess neointimal hyperplasia, and by immunofluorescent staining with mouse anti‐CD31 (ab64543, Abcam), rabbit anti‐eNOS (PA5‐16887, Thermo Fisher Scientific) and mouse anti‐alpha smooth muscle actin (*α*‐SMA, Abcam, ab7817) to assess re‐endothelialization and circumferential alignment of VSMCs, respectively. The detailed procedures for gene expression, en face staining and histological analysis of injured femoral arteries were available in the Supporting Information.

##### The Targeted Binding of DPP‐hADSCs to Balloon‐Injured Human Femoral Arteries

Human tissue samples were obtained from the Department of Vascular Surgery, Tianjin First Central Hospital (Tianjin, China). Informed consent was obtained from a donor (33 year old male, Asian). All studies using human samples were approved by the Donation Service of Tianjin First Central Hospital and Tianjin First Central Hospital's clinical research ethics committee (approval number2019N124KY), which has regulations consistent with the Helsinki Declaration. Bilateral balloon‐mediated human femoral arteries injury was performed by a vascular surgeon. The detailed procedure of operation is available in the Supporting Information. After the operation was completed, both injured human femoral arteries (≈10 cm in length per artery) were immediately harvested and each was cut into four equal samples. The samples were randomly divided into hADSCs group or 5 × 10^−6^ m DPP‐hADSCs group. Two samples were linked in series on one channel of the dual channel flow culture bioreactor. Firefly luciferase gene transfected hADSCs (Luc‐hADSCs) were obtained by the same method as rat ADSC gene transfection. A peristaltic roller‐pump placed distal to the vessels was used to pump culture medium from two three‐port glass fluid reservoirs, which contain 20 mL of DPP‐modified and unmodified Luc‐hADSCs suspension (10^5^ cells mL^−1^) respectively, via silicone tubes to the two chambers in parallel position. Flow rate was set as 12.41 cm s^−1^, which was within the range of human coronary blood flow velocity (9.9 ± 3.5 cm s^−1^).^[^ ^58^ ^]^ After culturing for 1 h at 37 °C and 5% CO_2_, the injured arteries were harvested and placed into 12 well plates. A total of 500 µL D‐Luciferin solution (150 µg mL^−1^ in PBS) was added. The BLI signal of Luc‐hADSCs was tested to evaluate the target potential of DPP‐hADSCs to injured human femoral arteries. After BLI testing, the samples were fixed with 4% paraformaldehyde and embedded in OCT for frozen cross sections. The sections were immunofluorescent stained with FITC anti‐human CD90 (BioLegend, 328107) and anti‐P‐selectin (sc‐8419, Santa Cruz Biotechnology, USA) to assess the targeting property of DPP‐hADSCs to the injured human femoral arteries.

##### Statistical Analysis

GraphPad Prism software version 5.0 (San Diego, CA, USA) was used for statistical analysis. Single comparisons were carried out using a paired Student's *t*‐test. Multiple comparisons were performed using a one‐way ANOVA and Tukey's post hoc analysis. The minimum significance level was set at *p < 0.05, **p < 0.01, and ***p < 0.001. Data are expressed as the mean ± standard error of the mean.

## Conflict of Interest

The authors declare no conflict of interest.

## Supporting information

Supporting Information
