# Supplementary material for: Targeted Repair of Vascular Injury by Adipose‐Derived Stem Cells Modified with P‐Selectin Binding Peptide
Source: Adv Sci (Weinh). 2020 Apr 22;7(11):1903516. doi: 10.1002/advs.201903516 (PMC7284211; doi:10.1002/advs.201903516)
Supplement: Supplementary file 1 — Supporting Information [file ADVS-7-1903516-s001.pdf]

Copyright WILEY-VCH Verlag GmbH & Co. KGaA, 69469 Weinheim, Germany, 2020.

Supporting Information

Targeted Repair of Vascular Injury by Adipose-derived Stem Cells Modified with P-selectin Binding Peptide

*Hongyu Yan, Xingyan Mi, Adam C. Midgley, Xinchen Du, Ziqi Huang, Tingting Wei, Ruihua Liu, Tengzhi Ma, Dengke Zhi, Dashuai Zhu, Ting Wang, Guowei Feng, Ying Zhao, Weiye Zhang, Ju He, Meifeng Zhu, * Deling Kong, * and Kai Wang**

Experimental Section

Materials: Fluorescein isothiocyanate isomer (FITC) was obtained from Sigma-Aldrich (St. Louis, MO, US). Dulbecco's modified Eagle's medium (DMEM) mixed 1:1 with Ham's F-12 (DMEM/F12) and fetal bovine serum (FBS) were purchased from Gibco (Life Technologies, Carlsbad, CA, US). Cell Counting Kit-8 (CCK-8) was purchased from Beyotime Biotechnology Co., Ltd. (Shanghai, China). The Live/Dead Viability/Cytotoxicity Kit was purchased from Invitrogen (Life Technologies). PrimeScript RT reagent Kit with gDNA eraser was purchased from Takara Bio (Kusatsu, Shiga, Japan). Hieff qPCR SYBR green master mix was purchased from Yeasen Biotech Co., Ltd. (Shanghai, China). Dialysis membranes was purchased from Tianjin Tiannan Technology Co., Ltd. (Tianjin, China).

Preparation of DMPE-PEG-PBP (DPP): DMPE-PEG-MAL (30 mg, 0.006 mmol) and P-selectin binding peptide (PBP) (30 mg, 0.029 mmol) were dissolved in 2 mL of dimethylformamide (DMF) followed by dropwise addition of triethylamine to adjust to pH 8.0. The reaction mixture was stirred for 24 h at ambient temperature under N_2 atmosphere. The mixture was then dialyzed against deionized water using dialysis membrane (MWCO 3,000 Da) for three days, and then lyophilized to obtain the DMPE-PEG-PBP powder.

Animal use and study approval: Sprague-Dawley (SD) rats, GFP^+ SD rats (male, weight 250-280 g) were obtained from the Laboratory Animal Center of the Academy of Military Medical Sciences (Beijing, China). All animal experiments were approved by the Animal Experiments Ethical Committee of Nankai University and were in accordance with the NIH Guide for Care and Use of Laboratory Animals. The accreditation number of the laboratory is SYXK(Jin) 2019-0003 promulgated by Tianjin Science and Technology Commission. The accreditation number of the laboratory is SYXK(Jin) 2019-0003 promulgated by Tianjin Science and Technology Commission.

Rat Adipose-derived stem cells (rat ADSCs) isolation, cultivation and identification: SD rats (male, weight 250-280 g) were sacrificed by overdose injection of pentobarbital sodium and inguinal adipose tissues were extracted, washed several times in sterilized PBS and cut into small pieces. Minced tissue was then digested in Kreb's Ringer Buffer solution supplemented with 2 mg mL^{-1} collagenase I (Sigma-Aldrich), 1% Bovine serum albumin（BSA), 2 mM glucose, and 25 mM HEPES at 37 ℃ for 45 min. The tissue digest was filtered through a stainless-steel mesh and the mature adipocytes were removed following gravity separation. The remaining cells were washed twice and plated in T75 flasks in complete DMEM/F12 medium containing 10% FBS and 100 U mL^{-1} penicillin/streptomycin (Gibco, Life Technologies). The culture medium was changed every three days until cells reached confluence. Third passage cells were used for immunophenotype analyses, using flow cytometry (FCM) to assess CD31, CD34, CD45, CD29, CD44, CD90 and CD105 cell-surface expression.

The proliferation, adhesion, migration and gene expression of DPP modified rat ADSCs: Rat ADSCs modified with 1, 5 and 25 μM DPP for 10 min and unmodified rat ADSCs were used for proliferation analysis.

For proliferation analysis, unmodified and DPP-modified rat ADSCs were seeded in a 48-well plate at a density of 8,000 cells per well (n=5) and cultured in 300 μL complete DMEM/F12 medium at 37 °C in a 5% CO_2 humidified incubator. The medium was changed every 24 h. After culture for 1, 3 and 5 days, 30 μL of CCK-8 reagent was added to each well and incubated for 2 h. Next, 100 μL supernatant from each well was transferred to a 96-well plate and absorbance was measured at 450 nm using an iMark Bio-Rad Microplate Reader (Bio-Rad, Hercules, CA, US). At the corresponding timepoint, cells were stained with Live/Dead Double Staining Kit and fluorescent images were captured using an inverted Leica DMi8 fluorescence microscope (Leica, Wetzlar, Germany).

For adhesion analysis, unmodified and DPP-modified firefly luciferase-transfected rat ADSCs (Luc-rat ADSCs) were seeded on 48-well tissue culture plastic plates (TCPS) at 2×10^4 per well and cultured in 500 μL complete DMEM/F12 medium. After incubation at 37 °C for 1 h or 4 h, wells were rinsed with PBS to remove non-adhered cells and 100 μL D-Luciferin solution (150 μg mL^{-1} in PBS) was added. The signal of Luc-rat ADSCs was measured using an IVIS Luminar bioluminescence imaging (BLI) imaging system (Xenogen Corporation, Hopkinton, MA, US) to evaluate cell adhesion. Following BLI, adhered cells were immediately observed under inverted phase contrast microscopy (Leica). In addition, the adhesion of unmodified and DPP-modified Luc-rat ADSCs on collagen IV were analyzed by the same methods as described above. TCPS were coated with collagen IV according to a previously described procedure.[1] In brief, collagen IV solution (human placenta, Sigma-Aldrich) was diluted to 50 μg mL^{-1} using distilled deionized water. Each well of a 48-well plate was coated with 150 μg mL^{-1} of collagen IV in 100 μL, for 1 h at room temperature. The collagen IV solution was removed, and wells were washed 3 times with PBS to dissociate the uncoated collagen IV. Cell seeding, incubation, washing and adhesion analysis methods were performed as described above.

For gene expression analysis, total RNA was harvested from extracted from unmodified rat ADSCs and DPP-modified rat ADSCs (25 μM DPP, 10 min) with TRIzol reagent (Invitrogen, Life Technologies) following the manufacturer's instructions. Isolated total RNA was reverse transcribed using reverse transcriptase kit (Takara Bio). Quantitative real-time PCR (qRT-PCR) was performed on a CFX96 RealTime PCR System (Bio-Rad) using SYBR Green-based real-time detection method (Roche, Mannheim, Germany). The relative expression level of the mRNA of interest was expressed as $2^{-(\Delta\Delta CT)}$ and normalized to the expression of the β-actin (*Actb*) housekeeping gene. Primer sequences are listed in Table S1.

Migration experiments were performed in 24-well trans-well Falcon plates (BD Biosciences, Franklin Lakes, NJ, US) and using cell culture inserts incorporating PET membranes with 8 μm pore size. To each lower well, 500 μL of complete DMEM/F12 medium was added. After serum starvation for 12 h, rat ADSCs were harvested, modified and suspended in serum-free DMEM/F12 medium at 5×10^5 cells mL^{-1}. Two-hundred microliters of unmodified and modified rat ADSCs suspension was seeded onto the well inserts. After incubation for a further 14 h, migrated cells were fixed, stained, and counted.

Human umbilical vein endothelial cells *(HUVECs) activation and P-selectin overexpression*: HUVECs were activated by tumor necrosis factor-α (TNF-α) according to previous report.[2] Briefly, glass slides of appropriate size were placed in 48-well polystyrene (PS) plates. HUVECs were seeded on the glass slides and grown to confluence at 37 °C, 5% CO_2 and in a humidified incubator in Endothelial Cell Medium (ECM). Confluent cell monolayers were washed with PBS and activated by exposure to 500 μL ECM medium containing 10 ng mL^{-1} TNF-α for 6 h. Next, activated HUVECs were washed twice with PBS and fixed with 4%

paraformaldehyde at 37℃ for 15 min. To stain for intracellular antigens, 0.1% (v/v) Triton X-100/PBS was used to permeate the membrane before blocking with 5% normal goat serum (Zhongshan Golden Bridge Biotechnology, Beijing, China). Following three washes with 0.01 mᴍ PBS, cells were incubated in 5% normal goat serum for 45 min at 4 °C and then incubated with anti-P-selectin antibody (1:50, Santa Cruz Biotechnology, Santa Cruz, CA, US) overnight at 4 °C. Following five washes with 0.01 mᴍ PBS, AlexaFluor-594 goat anti-mouse IgG (1:200, Invitrogen, Life Technologies) and phalloidin-AlexaFluor-488 (1:200, Invitrogen, Life Technologies) were applied for 2 h at room temperature, under darkness. The nuclei were counterstained with 4′,6-diamidino-2-phenylindole (DAPI) containing mounting solution (DAPI-Fluoromount-G, Southern Biotech, England, UK). Next, glass slides were removed from the plates and observed under a Zeiss Axio Imager Z1 fluorescence microscope (Zeiss, Oberkochen, Germany).

Wire-mediated femoral artery injury in rats: Following anesthesia by intraperitoneal injection of pentobarbital sodium (30 mg kg^{-1} body mass) and tail vein injection of anticoagulant heparin (100 units kg^{-1}), femoral arteries were exposed by longitudinal groin incision and monitored under a surgical microscope. To interrupt blood flow, a single knot was tied using 5-0 silk thread around the femoral artery proximal to the inguinal ligament, and another knot was tied using 7-0 silk thread around the femoral artery distal to the deep femoral branch. An incision hole was created distal to the deep femoral branch and an angioplasty guide wire (0.38 mm diameter) was introduced into the femoral artery, passed in and out 3 times to an approximate length of 8 mm, and left in place for 1 minute to denude and dilate the artery. The wire was then removed, and the arteriotomy site was ligated with an 9-0 suture. The knots were subsequently untied to restore blood flow.

P-selectin expression of rat injured femoral arteries: P-selectin expression of rat injured femoral arteries was assessed by *En-face* immunofluorescence staining. Briefly, 10 min after rat or human femoral artery injury, vessels were harvested and fixed in 4% paraformaldehyde. Samples were axially cut to expose the inner surface. After three rinses with 0.01 mᴍ PBS, the samples were incubated in 5% normal goat serum for 45 min at 4 °C. Next, samples were incubated with anti-P-selectin antibody overnight at 4 °C. After further washes with 0.01 mᴍ PBS for five times, AlexaFluor-594 goat anti-mouse IgG (1:200; Invitrogen, Life Technologies) was applied for 2 h at room temperature, under darkness. The nuclei were counterstained with DAPI-containing mounting solution. The lumen surfaces of the injured femoral arteries were observed under a confocal laser scanning microscope. The healthy rat femoral arteries were used as controls and were stained with the same protocol.

The gene expression of rat injured femoral arteries at day 1 post surgery: The rat injured femoral arteries were harvest and washed three time with PBS. Total RNA was extracted with TRIzol reagent (Invitrogen, Life Technologies) following the manufacturer's instructions. Following reverse transcription, qRT-PCR was performed on a CFX96 RealTime PCR System (Bio-Rad) using SYBR Green-based real-time detection method (Roche). The relative expression level of the mRNA of interest was expressed as $2^{-(\Delta\Delta CT)}$ and normalized to β-actin, respectively. Bulge-loop miRNA RT-PCR Primer Sets (one RT primer and a pair of qRT-PCR primers for each set) were designed and purchased from RiboBio (Guangzhou, China). Primer sequences used are listed in Table S2.

Mepacrine and CD90 En-face immunofluorescence co-staining: At 1-day post-operation, injured rat femoral arteries from the different treatment groups were harvested and fixed with 4% paraformaldehyde. After rinsing with 0.01 mᴍ PBS for 3 times, the samples were incubated in 5% normal goat serum for 45 min at 4 °C. Next, samples were incubated with anti-CD90-PE (2 μg mL^{-1}) and mepacrine (10 mᴍ) for 90 min. The lumen surfaces of the injured rat femoral arteries were then observed under a confocal laser scanning microscope.

CD45 and CD90 En-face immunofluorescence co-staining: At 1-day post-operation, injured rat femoral arteries from the different treatment groups were harvested and fixed with 4% paraformaldehyde. After rinsing with 0.01 mM PBS for three times, the pieces were incubated in 5% normal goat serum for 45 min at 4 °C. Next, samples were incubated with anti-CD90-PE (2 μg mL^{-1}) and anti-CD45-FITC (2 μg mL^{-1}) for 90 min. The lumen surfaces of the injured femoral arteries were then observed under a confocal laser scanning microscope.

Histological analysis of rat injured femoral arteries: At 21-days post-operation, injured femoral arteries were fixed in 4% paraformaldehyde at 4 °C overnight, then dehydrated in 30% sucrose solution until grafts sank to the bottom of the container. After embedding in OCT, explants were cut into 6 μm sections. Next, sections were stained with Verhoeff-Van Gieson (VVG). Images were observed under upright microscopy (Leica DM3000). For immunofluorescent staining, frozen sections were fixed in acetone at -20°C for 10 min, air-dried, and rinsed once with 0.01 mM PBS. Next, slides were incubated in 5% normal goat serum (Zhongshan Golden Bridge Biotechnology) for 45 min at 4 °C. To stain intracellular antigens, 0.1% (v/v) Triton-X-100/PBS was used to permeate membranes before incubation with blocking serum. The sections were incubated with the following primary antibodies overnight at 4°C: Mouse anti-CD31 (1:100; Abcam), rabbit anti-eNOS (1:100; Abcam), mouse anti-α-SMA (1:100; Abcam). After five rinses with PBS, AlexaFluor594 or 488 goat anti-mouse IgG (1:200; Invitrogen, Life Technologies) was applied for 2h at RT, under darkness. Nuclei were counterstained with DAPI-containing mounting solution. Sections without primary antibody incubations were used as negative controls. Slides were observed under fluorescence microscopy (Zeiss Axio Imager Z1), and images were acquired with a digital camera (AxioCam-MRm, Zeiss).

Differentiation and of ADSCs: ADSCs isolated from GFP^+SD rats were cultured as described above. Fourth passage GFP^+ rat ADSCs were modified with DPP (5 μM, 10 min). After wire-mediated rat femoral artery injury was induced, SD rats randomly received 2×10^6 GFP^+ rat ADSCs (ADSCs group), 5 μM DPP-GFP^+ rat ADSCs (5μM DPP-ADSCs group) or saline (untreated group). Treatments were administered in a volume of 200 μL saline solution containing 5 UI mL^{-1} heparin. Injection was performed as described above. Each group included 5 rats. At 21 days, injured vessels were harvested and fixed in 4% paraformaldehyde at 4 °C, overnight. Next, vessels were dehydrated in 30% sucrose solution until the grafts sank to the bottom of the container. Following embedding in OCT, explants were cut into 6 μm sections. Sections were co-stained with anti-GFP and anti-CD31 to evaluate the differentiation of GFP^+ rat ADSCs toward endothelial cell (EC) lineage. In addition, sections were co-stained with anti-GFP and anti-α-SMA to evaluate differentiation of GFP^+ rat ADSCs toward mesenchymal vascular smooth muscle cell (VSMC) lineages.

In vivo tracking of ADSCs localized to injured vessel： GFP^+ rat ADSCs were used for *in vivo* tracking of ADSCs localization to injured vessels. After wire-mediated femoral arteries injury was induced, SD rats randomly received 2×10^6 GFP^+ rat ADSCs (ADSCs group), 5 μM DPP-GFP^+ rat ADSCs (5μM DPP-ADSCs group) or saline (untreated group). Treatments were administered in a volume of 200 μL saline solution containing 5 UI mL^{-1} heparin. Injection was performed as described above. The rat injured femoral arteries were harvested at different time points (1, 4, 7, 14, 21 days after surgery, n=5 of each group) and fixed in 4% paraformaldehyde. Vessels were axially cut to expose the inner surface. After three rinses with 0.01 mM PBS, the sample were incubated in 5% normal goat serum for 45 min at 4 °C. Next, samples were incubated with anti-GFP antibody (Abcam, ab183734) overnight at 4 °C. After rinsing with 0.01 mM PBS for five times, AlexaFluor-488 goat anti-rabbit IgG (1:200; Abcam, ab150077)

was applied for 2 h at room temperature, under darkness. The nuclei were counterstained with DAPI-containing mounting solution. The lumen surfaces of the injured femoral arteries were observed under a confocal laser scanning microscope.

Assessment of ADSCs effects on activated HUVECs: The experiments were performed in 6-well Trans-well plates (BD Biosciences) containing cell culture inserts with PET membranes (0.4 μm pore size). HUVECs were seeded on lower wells at 4×10^5 per well and grown to confluence in ECM medium. DPP-modified (5 μM, 10 min) rat ADSCs were suspended in complete DMEM/F12 medium at 10^5 cells mL^{-1}. Confluent HUVECs were activated by TNF-α, as described above, then 1 mL of DPP-rat ADSCs suspension was added to each of the upper wells. After 24 h of co-culture, gene expression of activated HUVECs was assessed by qRT-PCR. Primer sequences are listed in Table S3.

After co-culture for 24 h, intracellular NO levels were detected using 3-Amino,4-aminomethyl-2',7'-difluorescein, diacetate (DAF-FM DA) fluorescence assay kit, according to the manufacturer's instruction (Beyotime Biotechnology). Nuclei were counterstained with DAPI-containing mounting solution. NO production was visualized by fluorescent detection using a confocal laser scanning microscope with excitation and emission wavelengths set at 495 and 515 nm, respectively. Inactivated HUVECs and activated HUVECs without co-culture were as positive and negative control groups, respectively.

Evaluation of DPP–ADSCs shielding from leukocyte and platelet adhesion: Experimental diagrams are shown in Figure S12. Glass slides of appropriate sizes were placed in 48-well PS plates (n=5). Following HUVEC confluence and activation by TNF-α (as above), 500 μL of unmodified or modified rat ADSCs suspension (4×10^4 cells mL^{-1}) were added to each well and incubated with shaking at 37 °C. Leukocytes were isolated from human peripheral blood purchased from the Tianjin Blood Center (Tianjin, China) using density gradient centrifugation. Unbound rat ADSCs were removed by washing, before 500 μL DiI-labelled leukocytes (2×10^5 cells mL^{-1}) were added to the activated HUVEC/rat ADSCs co-culture, for 1 h of further incubation. Unbound leukocytes were removed by washing and bound leukocytes were detected by DiI fluorescence under a fluorescence microscopy (Zeiss Axio Imager Z1). Leukocyte adhesion was determined as the average number of leukocytes per HPF (high power field).

Platelet shielding effect by 5 μM DPP-ADSCs was evaluated using the same method as with leukocytes. After unmodified or modified rat ADSCs were bound to activated HUVECs, 500 μL human platelet-rich plasma (PRP) was added to each well. After 1 h incubation, unbound platelets were removed by washing with PBS. Platelet adhesion was determined by mepacrine immunofluorescence staining as described above. Slides were observed under a confocal laser scanning microscopy. Platelet adhesion was determined as the average number of platelets per HPF. Five images per slide were averaged and five slides per group were assessed to obtain the final calculated values.

DPP-ADSCs inhibition of VSMC chemotaxis to activated platelets: Chemotactic experiments were performed in 24-well Transwell plates (BD Biosciences) with cell culture inserts incorporating PET membranes (0.8 μm pore size). Briefly, 450 μL human PRP was added to the lower well and 50 μL 0.5 mmol ADP solution was subsequently added to activate platelets. After incubation at 37 °C for 1 h, unattached platelets were removed by washing 3 times with PBS. Next, 500 μL of unmodified or DPP-rat ADSCs suspension (10^5 cells mL^{-1}) were added to each well and incubated with shaking at 37 °C. One hour later, unbound rat ADSCs were removed by washing 3 times with PBS and transferred to be cultured in 500 μL of serum-free DMEM/F12 medium. After serum starvation for 12 h, VSMCs (A10) were harvested with trypsin and resuspended in serum-free DMEM medium at 5×10^5 cells mL^{-1}. Next, 200 μL of

VSMC suspension was seeded onto the upper cell culture inserts. After incubation for 12 h, migrated cells were fixed, stained, and counted.

Balloon-injured human femoral artery: Human samples used in this study were obtained from the Department of Vascular Surgery, Tianjin First Central Hospital. Written informed consent was obtained from an organ donor (33 year old male, Asian). All studies using human samples were approved by the Donation Service of Tianjin First Central Hospital and Tianjin First Central Hospital clinical research ethics committee (approval number 2019N124KY), which has regulations consistent with the Helsinki declaration. The donor agreed to contribute all organs and tissues to scientific research or organ transplant. In line with the principle of priority access to vital organs, the heart, kidney, liver and lungs were harvested prior to balloon-injured human femoral artery. The vascular system of the donor was apparently healthy and vital organ operations had no influence on femoral artery integrity. The bilateral femoral arteries, of approximate length 10 cm, were isolated. A 6×15 mm balloon dilatation was inserted into each femoral artery. The balloon was inflated to 10-12 atm and then slowly pull out from the femoral artery under rotation. The whole process lasted at least 2 min. This procedure was performed a total of three times to inflict endothelium denudation and barotrauma. The bilateral femoral arteries were then harvested and immediately used to test the targeted binding of DPP-human ADSCs (hADSCs) in a flow culture bioreactor or for immunostaining.

P-selectin expression of human injured femoral arteries: P-selectin expression of human injured femoral arteries was tested by *En-face* immunofluorescence staining. Briefly, 10 min after human femoral artery injury, the vessels were harvested and fixed in 4% paraformaldehyde. Samples were axially cut to expose the inner surface. After three rinses with 0.01 mM PBS, the sample were incubated in 5% normal goat serum for 45 min at 4 °C. Next, samples were incubated with anti-P-selectin antibody overnight at 4 °C. After rinsing with 0.01 mM PBS for five times, AlexaFluor-594 goat anti-mouse IgG (1:200; Invitrogen, Life Technologies) was applied for 2 h at room temperature, under darkness. The nuclei were counterstained with DAPI-containing mounting solution. The lumen surfaces of the injured femoral arteries were observed under a confocal laser scanning microscope. Healthy, uninjured sections of human femoral artery were used as controls and stained according to the same protocol.

P-selectin overexpression in mice myocardial infarction area: Adult wildtype FVB mice (8-10 weeks old) were purchased from the Laboratory Animal Center of the Academy of Military Medical Science (Beijing, China). Mice were anesthetized through inhalation of isoflurane (1%-1.5%) with incubation and mechanical ventilation using a Hallowell EMC MicroVent 1 veterinary ventilator (Hallowell EMC, Pittsfield, MA, US). Once mice had entered a condition without breath depression and dysphoria, the left anterior descending coronary artery (LAD) was permanently ligated using a 7-0 silk suture via a left thoracotomy at the fourth intercostal rib. Infarction was considered successful by visualizing pale discoloration and an ST elevation on electrocardiograms. Afterwards, the chest wall and skin were closed and sutured. One day after myocardial infarction, mice were sacrificed and heart samples were harvested, fixed with Carnoy’s fixative solution, embedded in paraffin and cut into 6 μm sections. Anti-P-selectin antibody (1:50; Santa Cruz Biotechnology) diluted in blocking serum was added dropwise to cover heart sections, which were then incubated overnight at 4 ℃. Sections were then washed three times with PBS and secondary HRP-ligated goat anti-mouse antibody (1:500; Nanjing Bioworld Biotech, Nanjing, China) was added dropwise to cover sections and incubated at room temperature for 1 h, under darkness. After washing with PBS, diaminobenzidine (DAB) was used as a sensitive chromogen for colorization. Nuclei were counterstained with hematoxylin. Sections were observed under a Leica DMRXA2 microscope.

P-selectin overexpression on the lumen of vascular grafts: Electrospun Poly(ε-caprolactone) (PCL) grafts were fabricated and implanted into rat abdominal aortas according to previous reports.[3] Following implantation for 1 h, PCL grafts were explanted and rinsed with 0.9% NaCl solution. The explanted graft was longitudinally cut into two pieces. The lumen surface of the grafts was stained and observed using the same method as described above.

Supplementary Figures

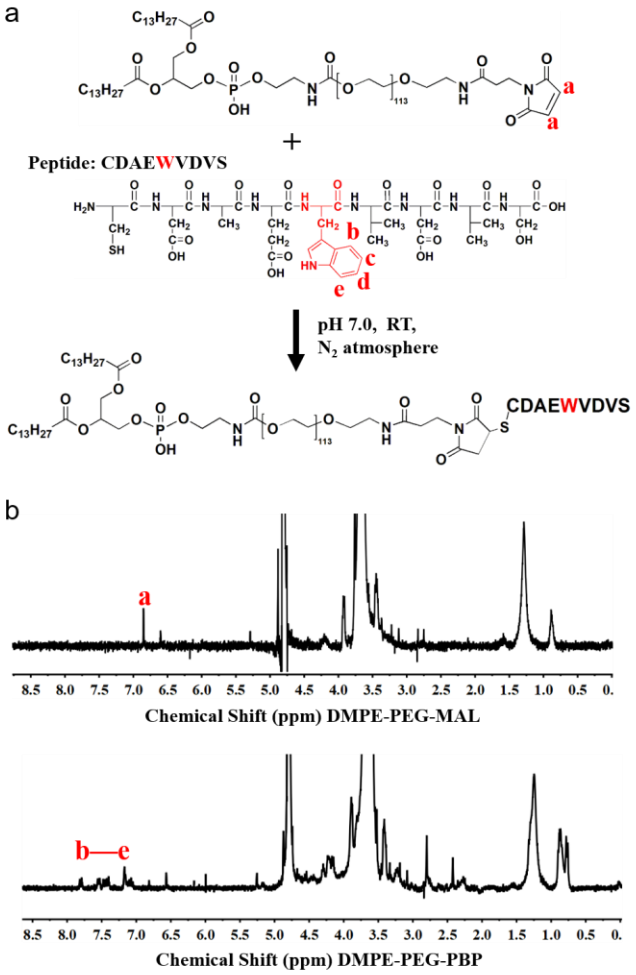

FigureS1. The synthesis and characterization of DMPE-PEG5000-PBP. (a) Schematic illustration of the conjugating reaction between DMPE-PEG5000-Maleimide and Cysteine-terminated peptides. ^{1}H-NMR spectra of DMPE-PEG5000-Maleimide (b) and DMPE-PEG5000-PBP in D_2O.

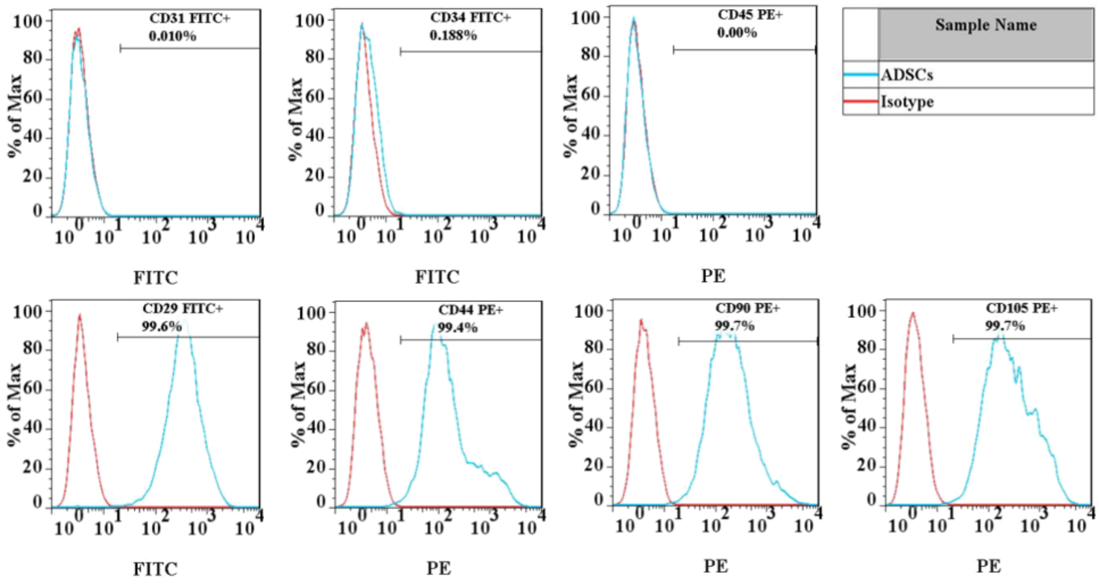

Figure S2. The FCM identification of third passage rat ADSCs (n=3).

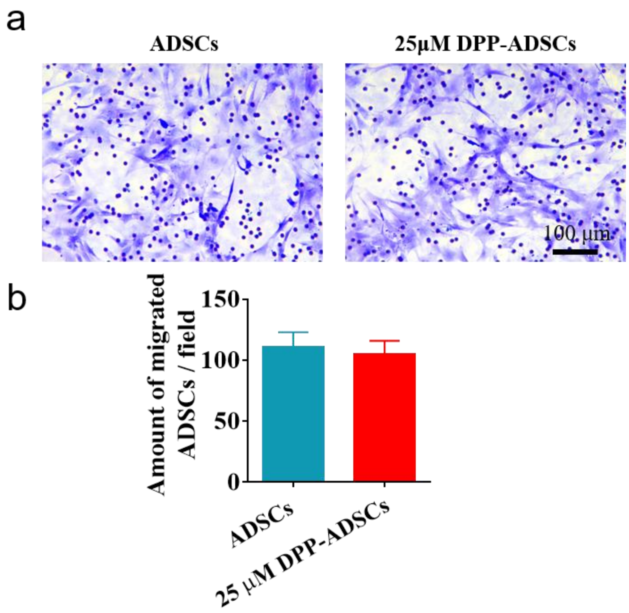

Figure S3. The effect of DPP modification on ADSCs migration. (a) Representative images of the trans-well migration assay of ADSCs and 25 μM DPP-ADSCs after culture for 14 h (n=3). (b) Quantitative analysis of the migrated ADSCs per microscopic field from the two groups.

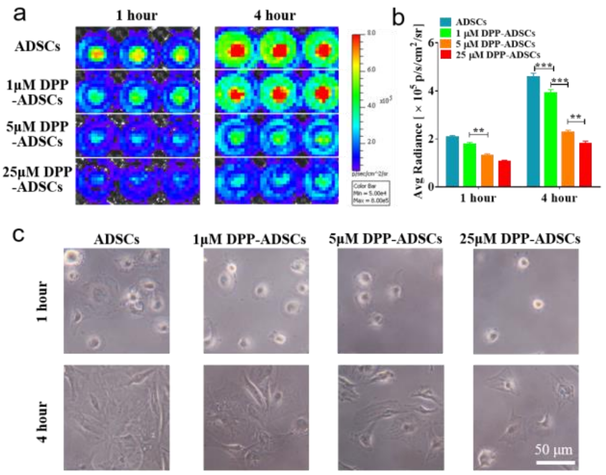

Figure S4. The adhesion of unmodified and DPP-Luc-ADSCs on collagen IV-coated TCPS. (a) The adhesion of unmodified and DPP-Luc-ADSCs was observed by BLI after incubation for 1 and 4 h (n=4). (b) The quantitative analysis of unmodified and DPP-Luc-ADSCs adhesion, based on the Luc fluorescence intensities. (c) The phase-contrast microscopy showed that there was no difference in cell morphology of unmodified and DPP-ADSCs adhered collagen IV coated TCPS on the after 1 h and 4 h incubation (n=4).

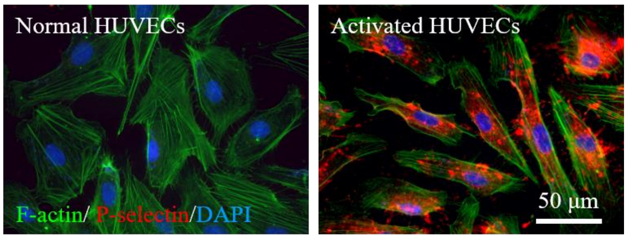

Figure S5. Immunofluorescence staining of P-selectin and F-actin in normal HUVECs and TNF-α activated HUVECs (n=3).

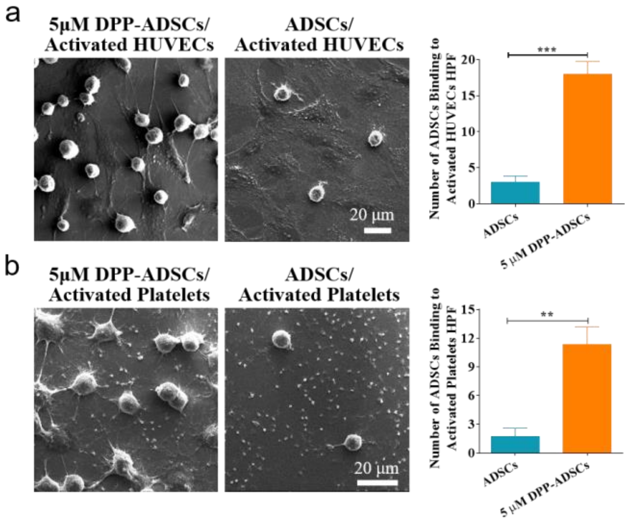

Figure S6. The number of unmodified ADSCs and 5 μM DPP-ADSCs binding to activated HUVECs (a) and platelets (b) was calculated based on SEM images. Three images per sample and five samples per group were used to obtain the calculation.

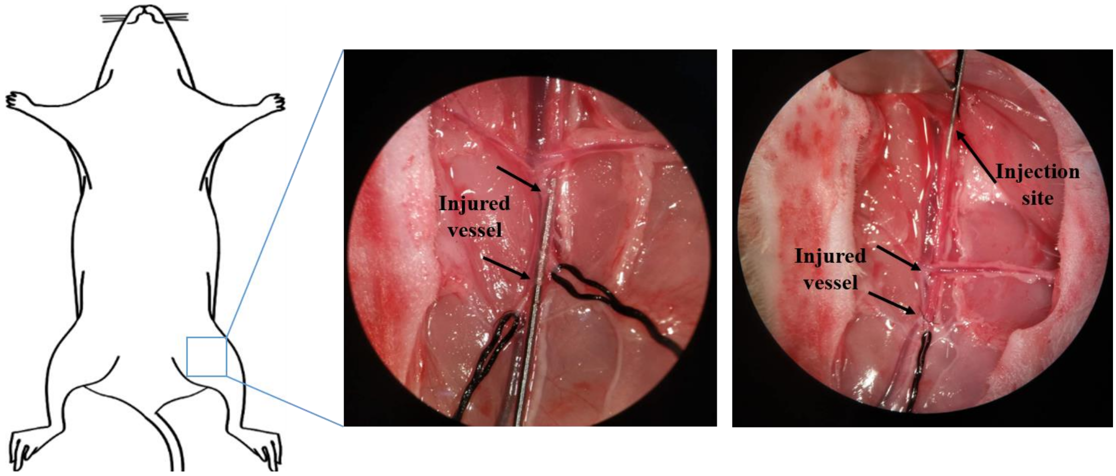

Figure S7. Schematic diagram of wire-mediated rat femoral artery injury and ADSCs injection. This cell delivery approach simulates the clinically used catheter-based cell delivery method, which are performed extensively and can easily integrated into PCI procedures.

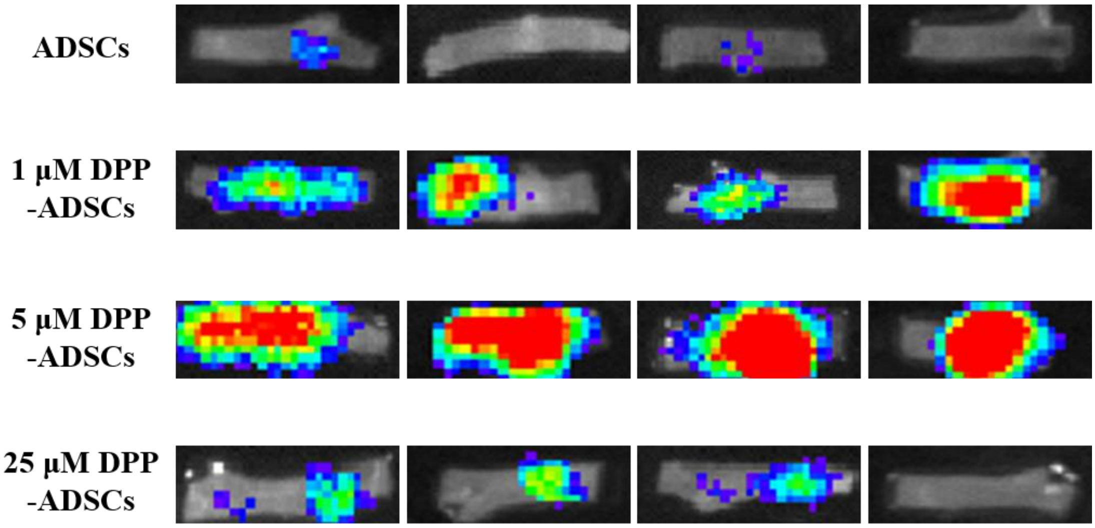

Figure S8. The in vivo targeting properties of DPP-ADSCs. Ten minutes after wire-mediated rat femoral artery injury, DPP-modified and unmodified Luc-ADSCs were systemically injected in rats. Ten minutes after injection, the targeted binding of DPP-modified and unmodified ADSCs to injured femoral arteries was observed by BLI. Four parallel samples were included in each group (n=4).

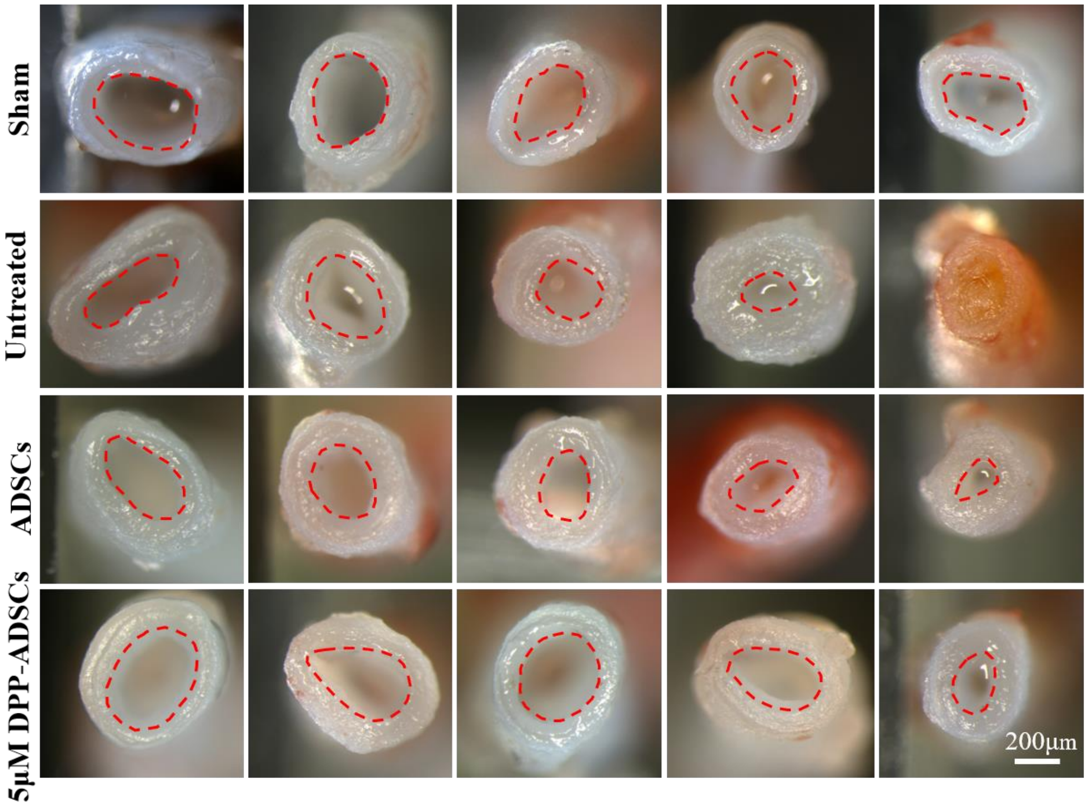

Figure S9. Stereomicroscope images of injured arteries in different treatment groups after 21 days. Five parallel samples were included in each group (n=5).

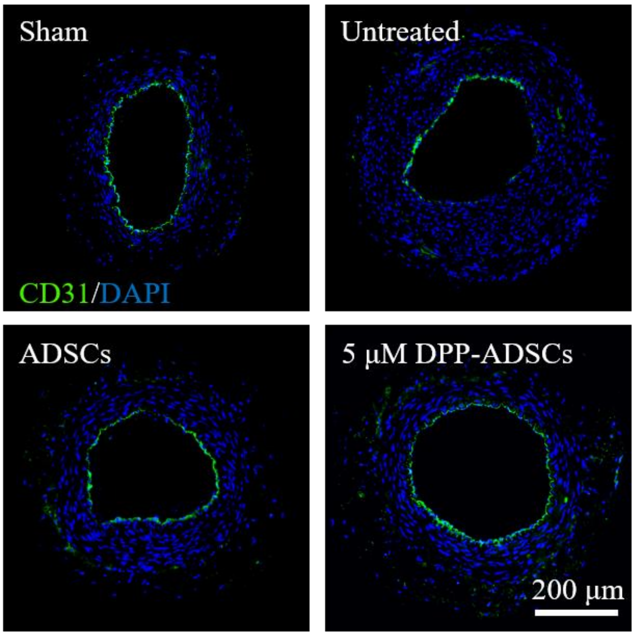

Figure S10. Re-endothelialization of injured arteries of the different treatment groups. Analyzed by anti-CD31 immunofluorescence staining at day 21 post surgery (n=5).

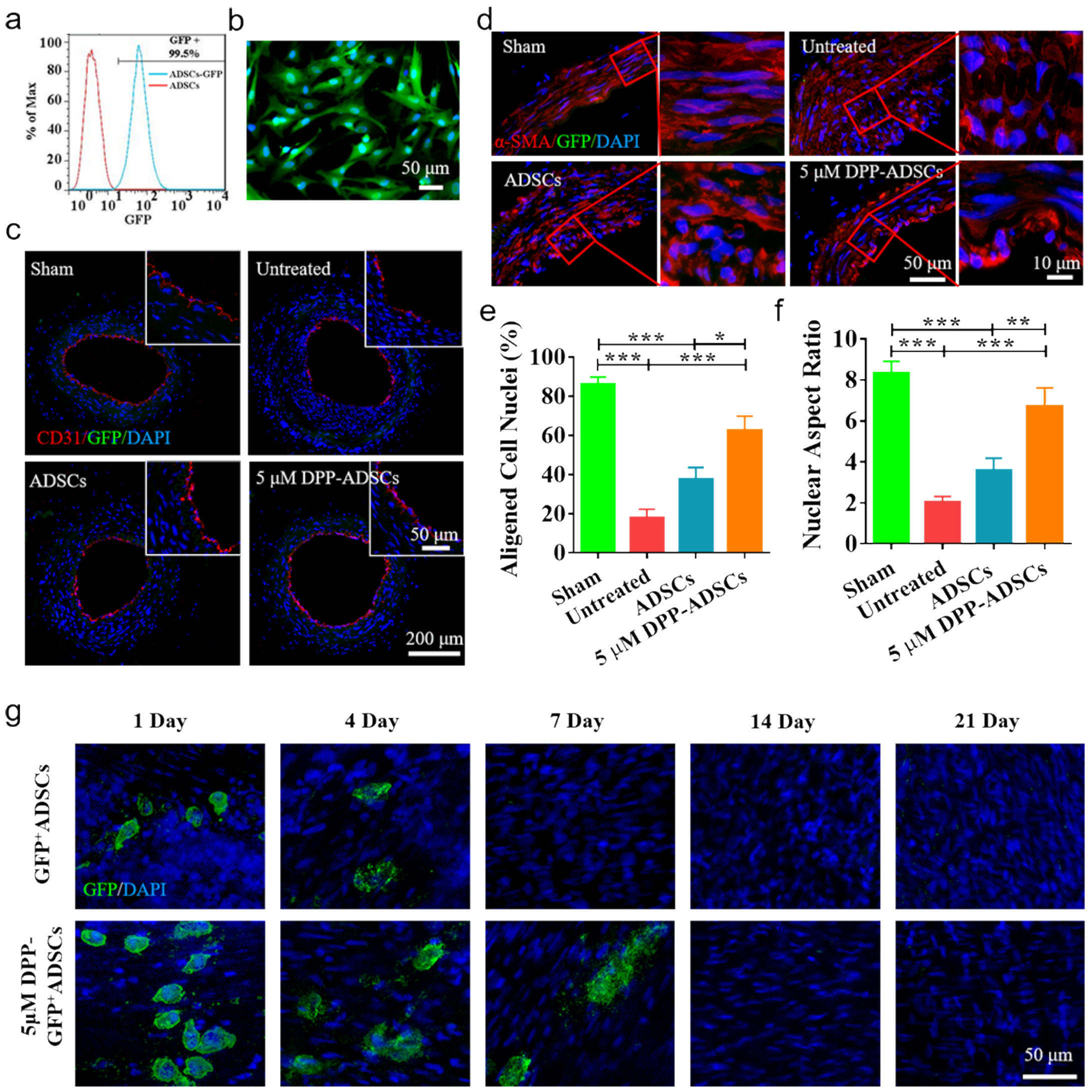

Figure S11. Assessment of ADSCs differentiation and residence at the vascular injury site. (a) FCM and (b) inverted fluorescence microscopy identification of GFP^+ADSCs. After 21 days of treatment with 5 μM DPP-modified or unmodified GFP^+ADSCs (n=5), the sections co-stained with anti-GFP and anti-CD31 showed no ADSCs differentiation toward ECs (c).The sections co-stained with anti-GFP and anti-α-SMA showed no ADSCs differentiation toward VSMCs (d). The circumferential alignment of VSMCs was observed by co-staining anti-GFP and anti-α-SMA, at 21 days after treatment. Right-hand images are enlargements of the red framed area of the left–hand images. Quantitative analysis of (e) aligned cell percentage and (f) nuclear shape index of VSMCs based on co-stained images. (g) The lumen surface of injured rat femoral arteries was observed by confocal microscopy to assess the GFP^+ADSCs and 5 μM DPP-GFP^+ADSCs residence at different time point (n=5).

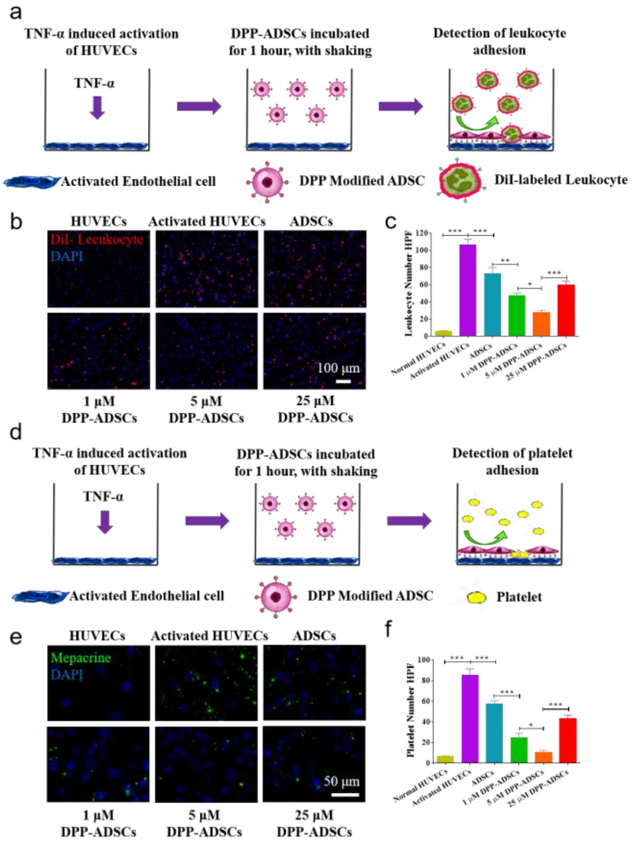

Figure S12. Shielding effect of ADSCs against adhesion of platelets and leukocytes to activated HUVECs. (a) Schematic illustration of the leukocytes shielding experiment. (b) The target binding of 5 μM DPP-ADSCs to activated HUVECs showed the most effectively shielding effect for the adhesion of DiI-leukocytes to activated HUVECs (n=4). (c) Quantitative analysis of the leukocytes adhesion of per high power field from each group. (d) Schematic illustration of the platelets shielding experiment. (e) The target binding of 5 μM DPP-ADSCs to activated HUVECs showed the most effectively shielding effect for the adhesion of platelets to activated HUVECs (n=4). (f) Quantitative analysis of the platelets adhesion of per high power field from each group.

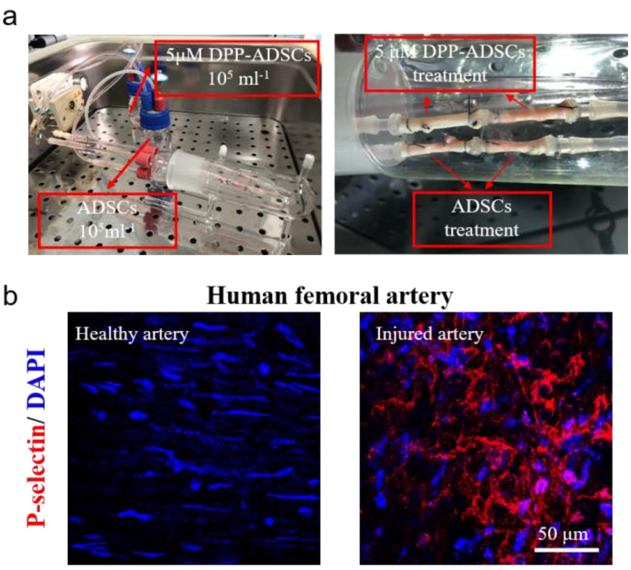

Figure S13. Dynamic culture system for testing the targeting of 5 μM DPP-modified and unmodified hADSCs to injured human femoral arteries (n=4). (a) Photograph of dynamic culture system. (b) P-selectin En-face staining of balloon injured human femoral arteries.

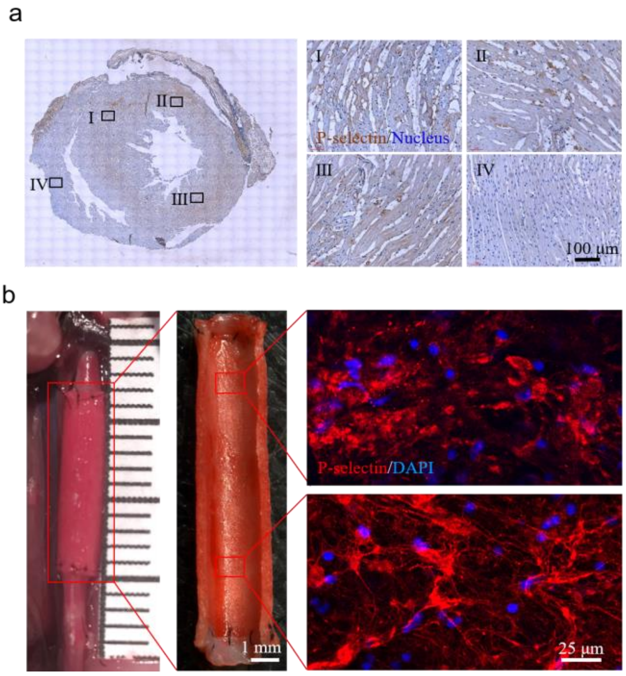

Figure S14. The P-selectin overexpression within myocardial infarction (MI) area and on the lumen surface of vascular graft. (a) Immunohistochemical staining showed that there were lots of P-selectin expression within MI area (Ⅰ, Ⅱ and Ⅲ), while almost no P-selectin expression within non-MI area (Ⅳ) after 1 day of MI. (b) After implantation in rat abdominal aorta for 1 h, lots of P-selectin had been observed on the lumen surface of electrospun PCL grafts by En-face immunofluorescence staining (n=3).

Supplementary Tables

Table.S1. Primers used for real-time PCR to detect the effect of surface modification on gene expression of rat ADSCs.

	Forward primer (5' to 3')	Reverse primer (5' to 3')
rat-Casp3	TTGGAACGAACGGACCTGTG	CGGGTGCGGTAGAGTAAGC
rat-P53	CACCTCCACACCTCCACCTG	TCCCGTCCCAGAAGATTCCC
rat-Bcl2	GAGTACCTGAACCGGCATCT	GAAATCAAACAGAGGTCGCA
rat-Bax	ATGGGCTGGACACTGGACTTC	GAGTGAGGCAGTGAGGAC
rat-Angpt1	GAAAATTATACTCAGTGGCTGCAAAAA	TTCTAGGATTTTATGCTCTAATAAACT
rat-Vegfa	TCACCGGAAAGACCGATTAAC	CCCTTCATGTCAGGCTTTCT
rat-Cxcl12	TTTCACTCTCCGTCCACCTC	ATCTGAAGGGCACAGTTTGG
rat-Il10	CAGAAATCAAGGAGCATTTG	CTGCTCCACTGCCTTGCTTT
rat-Hgf	TACACTCTTGACCCTGACACCC	TTTCCCATTGCCACGATAACA
rat-Actb	GTCGTACCACTGGCATTGTG	CTCTCAGCTGTGGTGGTGAA

Table.S2. Primers used for real-time PCR to detect the gene expression of rat injured femoral arteries with different treatment.

	Forward primer (5' to 3')	Reverse primer (5' to 3')
rat-Selp	AATGAAATCGCTCACCTC	TTATTGGGCTCGTTGTCT
rat-Il1b	GGATGATGACGACCTGC	CTTGTTGGCTTATGTTCTG
rat-Il6	CACAAGTCCGGAGAGGAGAC	TCACAAACTCCAGGTAGAAACG
rat-Il10	CAGAAATCAAGGAGCATTTG	CTGCTCCACTGCCTTGCTTT
rat-Hgf	TACACTCTTGACCCTGACACCC	TTTCCCATTGCCACGATAACA
rat-Vegfa	TCACCGGAAAGACCGATTAAC	CCCTTCATGTCAGGCTTTCT
rat-Actb	GTCGTACCACTGGCATTGTG	CTCTCAGCTGTGGTGGTGAA

Table.S3 Primers used for real-time PCR to detect the gene expression of activated HUVECs co-culturing with or without rat ADSCs.

	Forward primer (5’ to 3’)	Reverse primer (5’ to 3’)
human-CASP3	ACATGGCGTGTCATAAAATACC	CACAAAGCGACTGGATGAAC
human-P53	CCCAGCCAAAGAAGAAACCA	TTCCAAGGCCTCATTCAGCT
human-SELP	CAGGATCTCCCAGTTCCAAA	CTGAGGGCTTAGCAAAGGTG
human-ICAM1	CCCATGAAACCGAACACA	GGCATATGTCTTCCACTCTG
human-VCAM1	CATGGAATTCGAACCCAAACA	GGCTGACCAAGACGGTTGTA
human-IL6	TTCCAAAGATGTAGCCGCCC	GTTGGGTCAGGGGTGGTTATT
human-CXCL8	CATACTCCAAACCTTTCCACC	AAACTTCTCCACAACCCTCTG
human -CCL2	CAAGCAGAAGTGGGTTCA	GGGAAAGCTAGGGGAAAATAAG
human -NOS3	CTTTGCTCGTGCCGTGGACA	GCCCTCGTGGACTTGCTGCT
human -ACTB	GCCGATCCACACGGAGTACT	CTGGCACCCAGCACAATG

References

[1] R. N. Palchesko, K. L. Lathrop, J. L. Funderburgh, A. W. Feinberg, *Sci. Rep.* **2015**, *5*, 7955.

[2] a) J. Kim, K. Lee, J. Kim, D. Lee, M. Park, S. Choi, W. Park, S. Kim, Y. K. Choi, J. Y. Hwang, *Free Radical Biol. Med.* **2017**, *104*, 185; b) T. Marc, B. M. Lola, L. Yuan, L. Esther, P. Stéphane, B. Francoise, D. T. Nathalie, H. Caroline, M. Rute, D. An, Billiau, *Blood* **2008**, *111*, 4096.

[3] K. Wang, Q. Zhang, L. Zhao, Y. Pan, T. Wang, D. Zhi, S. Ma, P. Zhang, T. Zhao, S. Zhang, *ACS Appl. Mater. Interfaces* **2017**, *9*, 11415.
